# *Lactobacillus casei* bacterial ghost-based DNA vaccine enhances the immune protection effect by stimulating macrophages to regulate intestinal mucosal immunity

**DOI:** 10.3389/fimmu.2026.1839510

**Published:** 2026-07-08

**Authors:** Yuxin Wei, Xiaoli Yu, Xiayu Zhang, Hongzhe Zhao, Fei Teng, Guiwei Li, Wei Wu, Mingze Chen, Jiaxuan Li, Yanping Jiang, Wen Cui, Yingying Ma, Wenlong Zhang, Xinyuan Qiao

**Affiliations:** 1 Heilongjiang Key Laboratory for Animal Disease Control and Pharmaceutical Development, Department of Preventive Veterinary, College of Veterinary, Northeast Agricultural University, Harbin, China; 2 College of Animal Science and Veterinary Medicine, Heilongjiang Bayi Agricultural University, Daqing, China; 3 Branch of Animal Husbandry and Veterinary, Heilongjiang Academy of Agricultural Sciences, Qiqihar, China

**Keywords:** DNA vaccine, intestinal mucosal immunity, *Lactobacillus casei* BGs-based, macrophage, PoRV

## Abstract

Porcine rotavirus is a major threat to the swine industry, yet effective vaccines are still lacking. *Lactobacillus casei* bacterial ghosts (*L. casei* BGs) as DNA vaccine delivery vehicles represent a promising approach, providing a safe and structurally intact platform that enhances antigen presentation and elicits intestinal mucosal immunity. To elucidate how *L. casei* BGs regulate macrophage function and amplify mucosal immune responses, we investigated the response of macrophages and the intestinal mucosal immune system to an *L. casei* BG-based DNA vaccine encoding VP7. Using genetically engineered *L. casei* BGs, we performed *in vitro* macrophage stimulation and *in vivo* oral/intramuscular immunization in murine models, assessing phagocytic efficiency, TLR/MyD88/NF-κB pathway activation, M1 polarization, antigen presentation, serum IgG, fecal IgA, T cell proliferation, intestinal viral load, virus neutralization, and intestinal histopathology. Compared with a naked DNA vaccine, the *L. casei* BG-based vaccine significantly enhanced phagocytosis and antigen presentation and promoted M1 polarization via the TLR/MyD88/NF-κB pathway. Furthermore, oral administration of the vaccine proved superior to intramuscular injection in inducing mucosal immunity, preserving intestinal villus integrity, and enhancing antiviral protection. Collectively, this study establishes *L. casei* BGs as an effective DNA vaccine delivery system that enhances immunogenicity by modulating macrophage function and activating mucosal immunity, and offers a promising strategy for the control of porcine rotavirus infection.

## Introduction

Porcine rotavirus (PoRV), first identified in Australia in 1975 (1), has since become widespread and globally prevalent. In recent years, the detection rate, prevalence, and incidence of PoRV have shown a marked increase, with strain diversity becoming increasingly complex. In 2023, the detection rate of PoRV surpassed that of porcine epidemic diarrhea virus (PEDV) for the first time, making it the leading pathogen responsible for viral diarrhea in swines (1). VP7 is a rotavirus capsid protein that forms the outer capsid of the virion. It mediates cell entry by interacting with coreceptors and, together with VP4, represents a primary determinant of viral infectivity and a major target of neutralizing antibodies. VP7-specific neutralizing antibodies induced by VP7 vaccination or natural infection can provide protection against rotavirus infection and disease (2, 3). Although commercially available PoRV vaccines are already well established, they provide limited cross-protection. Current research is focused on developing promising candidate vaccines—such as DNA vaccines and mRNA vaccines—that can actively induce stronger immune memory and provide broader-spectrum protection.

DNA vaccines represent an innovative immunization strategy involving the direct delivery of plasmid DNA encoding specific antigens into host cells. This process enables host cells to produce target antigens endogenously, thereby inducing robust immune responses, including both humoral and cell-mediated immunity (4, 5). The development of DNA vaccines requires only the design and cloning of antigen-encoding genes, with diverse routes of administration, thereby eliminating the need for *in vitro* expression and purification of target proteins (6, 7). Unlike traditional vaccines, DNA vaccines lack intrinsic invasiveness and rely on passive uptake by host cells following administration. Subsequently, the antigens are expressed and presented by local antigen-presenting cells (APCs) (8). However, due to the low uptake efficiency and susceptibility of plasmid DNA to degradation, APCs often fail to effectively internalize, express, and present the antigen, resulting in weak immunogenicity and low levels of immune responses (9, 10). Therefore, the efficiency of antigen presentation is a key determinant of immune response. Enhancing APC-mediated antigen presentation, as well as improving antigen expression and immunogenicity, are critical factors influencing the efficacy of DNA vaccines.

Bacterial ghosts (BGs) are empty bacterial envelopes formed after the release of intracellular contents, while retaining intact cell wall and membrane structures as well as adhesion properties. They can act as a physical barrier, protecting internal antigens from degradation in the gastrointestinal tract (11, 12). As candidate vaccines or vaccine delivery systems, BGs are capable of inducing strong and effective humoral and cellular immune responses. In addition to the use of pathogenic BGs as promising vaccines, both pathogenic bacteria and probiotics can be converted into BGs to serve as efficient carriers for DNA vaccine delivery (13, 14). The use of BGs as DNA vaccine carriers significantly enhances uptake by macrophages and dendritic cells (DCs), thereby activating immunoregulatory pathways and strengthening host immune responses (15). To avoid safety concerns such as endotoxin production by Gram-negative bacteria, safer alternatives—including yeast and Gram-positive bacteria—are increasingly employed for the preparation of BGs for delivery purposes (16). In addition to ensuring the safety of the source organisms, it is also necessary to evaluate whether BGs administered via parenteral routes might induce autoimmune responses or immune tolerance. Studies have shown that pigs immunized with *Actinobacillus pleuropneumoniae* (APP) ghosts were effectively protected against APP, as confirmed by clinical, bacteriological, and serological analyses, without any observed adverse effects, demonstrating the safety and protective efficacy of BGs in parenteral immunization (17). Furthermore, this study evaluated the immunogenicity of BGs administered via multiple mucosal routes—including intragastric, oral, intranasal, intravaginal, intraocular, and rectal—and reported no evidence of immune tolerance.

Lactic acid bacteria are safe, non-toxic, and non-pathogenic Gram-positive probiotics, and are important components of the healthy gastrointestinal microbiota (18, 19). Some strains have been granted generally recognized as safe (GRAS) status and are widely used as oral vaccine delivery platforms. *Lactobacillus* casei bacterial ghosts (*L. casei* BGs) provide a relatively safe DNA vaccine delivery platform because they are derived from GRAS probiotic bacteria and exist as non-living bacterial envelopes incapable of replication or colonization while still retaining immunostimulatory activity (20). The ability of lactic acid bacteria to regulate intestinal mucosal immunity makes BGs derived from these organisms promising candidates for DNA vaccine delivery. BGs prepared from lactic acid bacteria retain key properties of the parent strains, including adhesion ability, acid resistance, and bile salt tolerance, enabling them to adhere to the intestinal mucosa and resist digestive degradation, thereby shortening their exposure time in digestive fluids and reducing the risk of degradation (21). Therefore, *L. casei* BGs are suitable for both parenteral and oral immunization.

Although lactic acid bacteria-derived BGs have demonstrated the ability to induce immune responses, comprehensive studies are still needed to elucidate how they enhance vaccine immunogenicity and host immune responses.

Toll-like receptors (TLRs) on macrophages recognize multiple Microbe-associated molecular patterns (MAMPs) on the surface of BGs, such as lipoproteins (LPP) and peptidoglycans (PGN), stimulating cytokine production that induces lymphocyte maturation and differentiation (22, 23). As key APCs, macrophages play indispensable roles in both adaptive and innate immunity. They defend against pathogens through phagocytosis and regulate immune responses by modulating lymphocyte activation and proliferation (24). Intestinal lamina propria macrophages are essential components of innate immunity, maintaining intestinal homeostasis and participating in antigen presentation (25, 26). Compared to other APCs, they exhibit stronger phagocytic and antigen-expressing capabilities, playing a critical role in sustaining intestinal homeostasis (27). Thus, effectively regulating intestinal macrophage activity is crucial for enhancing mucosal immune responses.

Recent advances in live bacterial vector-based platforms have further expanded the possibilities for oral vaccines. For example, Yue et al. recently reported an oral vaccine platform (BacOR-Fn-T+phiX174) featuring genetically encoded dual-antigen arrays and inducible bacterial lysis (28). The authors demonstrated promising safety outcomes in their preclinical models. Complementing such live platforms, our strategy employs a non-living bacterial ghost (BG) carrier to deliver antigen-encoding plasmids. By design, this approach avoids viability-related concerns, providing a different safety profile. However, the potential of *L. casei* BGs as a DNA vaccine delivery system for enhancing PoRV antigen presentation, and the underlying immunological mechanisms involving the modulation of intestinal macrophage function, have not been well characterized.

In this study, we aimed to address this gap. *L. casei* BGs were employed as DNA vaccine carriers to encapsulate pCI-PoRV-VP7 for targeted delivery. The immunomodulatory effects of DNA-loaded *L. casei* BGs on macrophage function were systematically evaluated through *in vitro* stimulation assays and ex vivo analysis of macrophages isolated from the intestinal tissues of immunized animals, thereby demonstrating their potential to enhance vaccine immunogenicity.

## Materials and methods

### Plasmids and animals

*Lactobacillus casei* American Type Culture Collection (ATCC) 393 was obtained from the Netherlands NIZO Food Research B.V. Institute (Ede, Netherlands). The pCI-PoRV-VP7 plasmid was constructed using the following procedures: Total RNA extracted from Porcine rotavirus (PoRV JL94 strain) was reverse-transcribed to cDNA, followed by PCR amplification of the VP7 gene. The amplified product was digested with Kpn I/Sal I (New England biolabs, Massachusetts, USA) and ligated into the corresponding sites of pCI-neo vector (Promega, Madison, WI, USA) to generate recombinant plasmid pCI-PoRV-VP7, which was verified by restriction enzyme analysis. The plasmid was amplified in *Escherichia coli* DH5α (*E. coli* DH5α) competent cells. Plasmid DNA was purified using rapid DNA extraction Kits (Sigma, St.Louis, MO, USA) and stored at -20 °C.

BALB/c mice (6–8 weeks old, female, specific pathogen-free) from Changsheng Biotechnology Co., Ltd (Liaoning, China) were housed under controlled conditions with free access to food and water after 7-day acclimatization. All experimental procedures were approved by the Heilongjiang Provincial Animal Care Committee.

BALB/c mice were selected in this study because they are a well-established model for evaluating DNA vaccine-induced mucosal immunity and macrophage-mediated immune responses, particularly in studies involving oral vaccine delivery and bacterial ghost-based vaccine platforms (29, 30).

### Construction of recombinant *L. casei* strain for ghost production

The recombinant strain pPG-2-hocb*/L. casei* 393 (31) was constructed and is preserved in our laboratory. No additional transformation of *L. casei* was performed in this study; the pre-established recombinant strain was used directly.

### Preparation of *L. casei* bacterial ghosts

This strain carries the holin gene *hocb*, derived from *Lactobacillus casei* phage Lcb. Upon induction, the gene encodes a hydrophobic membrane protein, holin, which localizes to the cell membrane. Holin proteins oligomerize at the mid-cell or at both poles, forming transmembrane pores with diameters ranging from approximately 40 to 400 nm (32). Driven by the osmotic pressure gradient between the intracellular and extracellular environments, intracellular contents (e.g., nucleic acids and proteins) passively diffuse through these pores. The cell envelope remains intact, preserving the original cell morphology and ultimately generating empty cell shells known as BGs (33). This lysis process can be regulated by culture conditions: plasmid stability is maintained under chloramphenicol selection, and expression of the *hocb* gene is induced by sucrose, enabling controlled preparation of bacterial ghosts. According to a previously described method (34), recombinant *L. casei* was cultured in MRS medium supplemented with 10 μg/mL chloramphenicol (MedChemExpress, USA) at 37 °C for 24* h*, and then induced with 2% fructose for 36* h*. BGs were harvested after OD600 stabilization and centrifuged at 6500 rpm (≈ 5,000 × g) for 20* min*. The pellets were treated with gentamicin (50 μg/mL) and streptomycin (100 μg/mL), washed with Phosphate-Buffered Saline (PBS), and stored at 4 °C.

### Loading of plasmid DNA into *L. casei* BGs and quantification of loading efficiency

Plasmid DNA (pCI-PoRV-VP7/pCI-EGFP) was loaded into *L. casei* BGs via passive diffusion through lysis-induced pores. Lyophilized BGs were rehydrated in buffer (100 mM NaCl, 10 mM sodium acetate, and 10 mM 4-(2-hydroxyethyl)-1-piperazineethanesulfonic acid, pH 7.5) containing 1 mg/mL plasmid cocktail. DNA binding was facilitated by adding 25 mM CaCl_2_, followed by vortexing at 37 °C for 15* min*. The loaded BGs were then centrifuged at 13000 rpm (≈ 10,000 × g) for 20* min*, washed with PBS, and resuspended in sterile PBS.

After plasmid loading and washing, the residual plasmid DNA in the bacterial ghosts was extracted using a plasmid extraction kit and quantified by SYBR Green real-time quantitative PCR. The qPCR primers were Pac-F (5’-ATAGTCGTACTTGCACCGCTC-3’) and Pac-R (5’-ACTGCGACAATGTTTCTGTCC-3’), amplifying a 193 bp fragment. A standard curve was generated using known concentrations of plasmid DNA, and the initial plasmid concentration in each sample was calculated based on the standard curve and the Ct values. The 20 μL reaction mixture contained: 10 μL of Mix buffer, 0.3 μL each of forward and reverse primers, 8.4 μL of nuclease-free water, and 1 μL of template DNA. The thermal cycling protocol was: 95 °C for 2 min, followed by 40 cycles of 95 °C for 15 s and 60 °C for 1 min. Each sample was run in quadruplicate, and a no-template blank control was included. Data acquisition and analysis were performed using the Bio-Rad CFX Manager real-time PCR detection system.

The BG suspension was lyophilized to obtain dry BG powder. The lyophilized BGs were collected and weighed using an analytical balance under dry conditions. The plasmid loading capacity was calculated as the amount of loaded plasmid DNA divided by the dry weight of lyophilized BGs and expressed as μg plasmid DNA/mg lyophilized BGs dry weight.

### Detection of loading stability of *L. casei* BGs

Lyophilized BGs powder (10 mg) were mixed with 50 μL of plasmid solution containing 1000 ng/μL DNA and incubated at room temperature to allow plasmid loading. After loading, the mixture was centrifuged at 12,000 rpm and the supernatant was discarded. The pellet was resuspended in 400 μL PBS for washing, and 100 μL of the suspension was taken as the first wash fraction. The remaining 300 μL was centrifuged again, the supernatant removed, and the pellet resuspended in 300 μL PBS for a second wash; 100 μL was taken as the second wash fraction. This procedure was repeated to obtain the third wash fraction. From each wash fraction, residual plasmid DNA retained in the bacterial ghosts was extracted using a plasmid extraction kit and quantified by SYBR Green real-time quantitative PCR.

### *In vitro* assessment of plasmid transfer to macrophages

Peritoneal macrophages were seeded in 6-well plates and treated with pCI-EGFP or pCI-EGFP-loaded BGs (5 × 10^7^ particles/mL) for 2 h. After washing with PBS to remove residual BGs, the cells were cultured at 37 °C for 48* h*. GFP expression was analyzed by fluorescence microscopy (Axio Observer Primo, Ulm, Germany), flow cytometry (FACSCalibur, BD Biosciences, Franklin Lakes, NJ, USA), and Western blot. In Western blot analysis, a specific band of approximately 62 kDa corresponding to the EGFP-VP7 fusion protein was detected using anti-GFP antibodies. Cell viability was assessed at 24 h using a Cell Counting Kit-8 (CCK-8) assay. Untreated cells served as controls.

### Observation by Transmission Electron Microscopy and Scanning Electron Microscopy

After induction, the bacterial cells were collected by centrifugation at 4,500 rpm (≈ 2,400 × g) for 15 min, and the pellet was washed three times with PBS. The pellet was resuspended in 1.5 mL of 2.5% glutaraldehyde in 0.1 M phosphate buffer (pH 6.8) and fixed at 4 °C. After fixation, the sample was rinsed with the same buffer, post-fixed with 1% osmium tetroxide, and rinsed again. The fixed sample was then dehydrated through a graded ethanol series of 50%, 70%, 90%, and 100% (v/v), followed by further dehydration with a mixture of 100% ethanol and 100% acetone, and finally with pure acetone.

For Transmission Electron Microscopy (TEM) observation: After dehydration, the sample was infiltrated and embedded in a mixture of pure acetone and embedding resin, and then polymerized at 60 °C for 3–6 days. After trimming of the embedded blocks, ultrathin sections of approximately 50–100 nm in thickness were cut using an ultramicrotome. The sections were double-stained with uranyl acetate and lead citrate. The internal morphology of bacterial ghosts was observed and photographed under a transmission electron microscope.

For Scanning Electron Microscopy (SEM) observation: After dehydration, the sample was subjected to CO_2_ critical−point drying to preserve the surface structure. The dried sample was mounted onto an aluminum stub using conductive adhesive tape and sputter−coated with a thin layer of gold (or platinum) to enhance conductivity. The surface morphology of bacterial ghosts was then observed and photographed under a scanning electron microscope.

### Cell viability assay

Cell viability was evaluated using a CCK-8 (Beyotime, Shanghai, China) according to the manufacturer’s instructions. Briefly, peritoneal or intestinal macrophages were seeded in 96-well plates and cultured at 37 °C in a humidified atmosphere containing 5% CO_2_ for 24* h*. Subsequently, 10 μL of CCK-8 solution was added to each well, and the plates were gently shaken and incubated at 37 °C for 2* h*. Color development was visually observed, and the reaction was terminated when obvious differences were observed among groups. The optical density (OD) at 450 nm was measured using a microplate reader (Berthold Technologies, Bad Wildbad, Germany). The cell proliferation rate was calculated as (A − C)*/*(B − C) × 100%, where A represents the OD at 450 nm of the treatment group, B represents that of the untreated control group, and C represents that of the blank well.

### Immunization design

BALB/c mice were randomly divided into twenty groups (n = 5 per group) and immunized via either oral or intramuscular injection. For some animal-derived endpoint analyses, a subset of biological samples was used, and the actual number of biological replicates included in each analysis is specified in the corresponding figure legend.

The specific immunization protocol and groups are shown in **Table 1**.

**Table 1:** Immunization and challenge.

Group	Route	Immunogen	Dose	Immunization days	PoRV challenge
1	oral	pCI-PoRV-VP7	50 µg per mouse	0 、14 、28	no
2	oral	*L. casei* BGS+pCI-PoRV-VP7	6.8 mg dry weight, containing 50 μg of plasmid per mouse	0 、14 、28	no
3	oral	pCI-PoRV-VP7-EGFP	80 µg per mouse	0 、14 、28	no
4	oral	*L. casei* BGS+pCI-PoRV-VP7-EGFP	13 mg dry weight, containing 80 μg of plasmid per mouse	0 、14 、28	no
5	oral	*L. casei* BGs	6.8 mg in PBS	0 、14 、28	no
6	oral	PBS (negative control)	200μL	0 、14 、28	no
7	oral	pCI-PoRV-VP7	50 µg per mouse	0 、14 、28	yes
8	oral	*L. casei* BGS+pCI-PoRV-VP7	6.8 mg dry weight, containing 50 μg of plasmid per mouse	0 、14 、28	yes
9	oral	*L. casei* BGs	6.8 mg in PBS	0 、14 、28	yes
10	oral	PBS (negative control)	200μL	0 、14 、28	yes
11	intramuscular	pCI-PoRV-VP7	50 µg per mouse	0 、14 、28	no
12	intramuscular	*L. casei* BGS+pCI-PoRV-VP7	6.8 mg dry weight, containing 50 μg of plasmid per mouse	0 、14 、28	no
13	intramuscular	pCI-PoRV-VP7-EGFP	80 µg per mouse	0 、14 、28	no
14	intramuscular	*L. caseiBGS* +pCI-PoRV-VP7-EGFP	13 mg dry weight, containing 80 μg of plasmid per mouse	0 、14 、28	no
15	intramuscular	*L. casei* BGs	6.8 mg in 50μL PBS	0 、14 、28	yes
16	intramuscular	PBS (negative control)	50μL	0 、14 、28	yes
17	intramuscular	pCI-PoRV-VP7	50 µg per mouse	0 、14 、28	yes
18	intramuscular	*L. casei* BGS+pCI-PoRV-VP7	6.8 mg dry weight, containing 50 μg of plasmid per mouse	0 、14 、28	yes
19	intramuscular	*L. casei* BGS	6.8 mg in 50μL PBS	0 、14 、28	no
20	intramuscular	PBS (negative control)	50μL	0 、14 、28	no

The immunization regimen consisted of three doses administered at 14-day intervals (i.e., days 0, 14, and 28). Unless otherwise specified, all endpoint analyses were performed 14 days after the final (third) immunization. Viral challenge was performed on day 14 after the final immunization, and samples for viral load and neutralization were collected 7 days post-challenge.

Serial serum and fecal samples were collected weekly from days 1 to 42 post-primary immunization. For fecal samples, 0.1* g* of each sample was homogenized in 400 µL of fecal lysis buffer, vortexed, and incubated overnight at 4 °C, followed by centrifugation at 9500 rpm (≈ 10,000 × g) for 10* min*. For serum preparation, blood samples were centrifuged at 4 °C and 6500 rpm (≈ 4,800 × g) for 20* min* to obtain serum. All samples were stored at −20 °C until analysis.

The jejunum of mice was collected at the endpoint. TRIzol reagent was added, and the samples were kept on ice, then placed on a magnetic vortex mixer and vortexed for 3 min. After centrifugation at 5000 rpm (≈ 3,000 × g) for 20 min, the supernatant was collected. The portion was used to detect specific IgA levels in mouse intestinal homogenates by Enzyme-Linked Immunosorbent Assay (ELISA).

Jejunum tissues were collected from mice at the endpoint. The intestinal supernatant samples were prepared as described previously. An aliquot of the sample was mixed with 5× SDS to a final concentration of 1× SDS, boiled at 100 °C for 10 min, and then subjected to protein gel electrophoresis followed by immunoblot analysis (see Immunoblot analysis section).

### Viral challenge

Fourteen days after the final immunization, mice in each group were orally challenged with 200 µL of PoRV (10^5^ TCID_50_/mL), corresponding to 2 × 10^4^ TCID_50_ per mouse.

### Isolation and identification of splenic T cells

Spleens were aseptically collected from either naive mice or mice euthanized at the endpoint. The spleens were gently homogenized and passed through a 70 μm cell strainer to obtain single-cell suspensions. The suspensions were carefully layered over an equal volume of lymphocyte separation medium and centrifuged at 400 × g for 20 min at room temperature. The lymphocyte-enriched interface was collected, washed twice with PBS, and resuspended in RPMI-1640 medium. The isolated splenocytes were analyzed by flow cytometry using CD3 staining to identify mature T cell populations.

### Preparation, transfection, and grouping of macrophages

Intestinal tissues were collected from orally or intramuscularly vaccinated BALB/c mice and placed in a Petri dish. After removal of luminal contents, the intestines were washed with PBS supplemented with penicillin (500 U/mL) and streptomycin (500 μg/mL) to minimize microbial contamination. The tissues were cut into small fragments and incubated in Hank’s balanced salt solution supplemented with 1 mM EDTA, 1 mM DTT, 1% penicillin-streptomycin and 5% serum at 37 °C for 40* min*. The intestinal fragments were further digested in a solution containing 1.5 mg/mL collagenase VIII (Proteintech, Wuhan, China), 40 μg/mL DNase I, and 5% serum at 37 °C for 30* min*. The resulting cell suspension was filtered through a 100-mesh cell strainer and centrifuged at 1000 rpm (≈ 110 × g) for 10* min*. The supernatant was discarded, and the cell pellet was resuspended in 4 mL of 50% Percoll solution. Subsequently, 2 mL of 80% Percoll solution was carefully layered onto the 50% Percoll solution. After centrifugation at 1200 rpm (≈ 160 × g) for 20* min*, cells at the interface were collected, yielding a population primarily composed of intestinal macrophages. Flow cytometry was used to detect the surface markers CD45 and CD64 on intestinal macrophages from mice. The antibodies used were rabbit anti-mouse CD45-FITC and CD64-PE (Thermo Fisher Scientific, Waltham, USA). In addition, intestinal macrophages isolated from immunized mice were collected, and their cell viability was assessed using a CCK-8 assay kit.

Peritoneal macrophages were isolated from naive, non-immunized BALB/c mice. Briefly, 5 mL of pre-chilled RPMI-1640 medium was injected into the peritoneal cavity of BALB/c mice. After gentle massage of the abdomen, the lavage fluid was aspirated using a syringe and centrifuged at 1300 rpm (≈ 1,000 × g) for 7* min* at room temperature. The cell pellet was resuspended in RPMI-1640 medium supplemented with 10% fetal bovine serum and cultured at 37 °C in a 5% CO_2_ incubator. After 6* h*, non-adherent cells were removed by replacing the medium, and the adherent cells were identified as peritoneal macrophages. Flow cytometry was used to detect the surface markers CD11b and F4/80 on murine peritoneal macrophages. The antibodies used were Alexa Fluor–conjugated rabbit anti-mouse CD11b and PE-conjugated rabbit anti-mouse F4/80 (Thermo Fisher Scientific, Waltham, USA).

Peritoneal macrophages were assigned to five groups: (i) cells treated with pCI-PoRV-VP7-loaded BGs at concentrations of 5 × 10^7^ cells/mL BGs and 750 ng/mL (pCI-PoRV-VP7); (ii) cells treated with pCI-PoRV-VP7 alone (750 ng/mL); (iii) cells stimulated with BGs alone (5 × 10^7^ cells/mL); (iv) cells stimulated with Lipopolysaccharide (LPS, 1 μg/mL) as a positive control; and (v) cells treated with an equal volume of PBS as a negative control.

### Enzyme-linked immunosorbent assay

Peritoneal and intestinal macrophages (1 × 10^6^ cells/well) were seeded in 12-well plates and pre-cultured for 12* h* at 37 °C. The cells were then treated with naked pCI-PoRV-VP7 plasmid (1 mg/mL), BGs (1000 bacteria/cell), or pCI-PoRV-VP7-loaded BGs (MOI = 1000) for 2* h* at 37 °C. After three washes with PBS, fresh medium was added, and the cells were further incubated. At 6, 12, 18, 24, and 30* h* post-treatment, the levels of IL-2, IL-6, IL-1β, IL-12, and TGF-β in the culture supernatants were measured using ELISA kits (Shanghai Meilian Biological Technology Co., Ltd., Shanghai, China) according to the manufacturer’s instructions. To evaluate T cell polarization, blood samples were collected from mice after the final immunization. The serum levels of IFN-γ, IL-2, IL-6, IL-17, and IL-22 in different immunization groups were determined using ELISA kits (Shanghai Meilian Biological Technology Co., Ltd., Shanghai, China) following the manufacturer’s instructions.

### Real-time PCR

Total RNA was extracted from macrophages in each treatment group using an RNeasy Mini Kit (Feijie, Shanghai, China) according to the manufacturer’s instructions. cDNA was synthesized from 1.0 μg of RNA using a reverse transcription kit (Takara, Dalian, China). To investigate macrophage-mediated recognition of microbial components, the mRNA expression levels of Toll-like receptors (TLRs; TLR1–TLR9) were evaluated. TLR1/2 and TLR2/6 heterodimers recognize bacterial lipopeptides and peptidoglycan, TLR4 recognizes LPS, and TLR5 recognizes flagellin, whereas TLR3, TLR7, TLR8, and TLR9 are primarily involved in sensing microbial nucleic acids, including bacterial DNA and viral RNA. Since BGs preserve diverse bacterial MAMPs and the vaccine construct additionally expresses the viral antigen VP7, it was necessary to profile the full spectrum of TLR1–TLR9 to comprehensively assess potential recognition pathways associated with both bacterial and viral antigenic components. Activation of these receptors triggers downstream signaling cascades, leading to the production of pro- and anti-inflammatory cytokines.

The mRNA levels of IL-2, IL-6, IL-12, TGF-β, IL-1β, TLR1–TLR9, MyD88, NF-κB, IL-10, NOS2, Arg-1, and TNF-α were quantified by real-time quantitative PCR (RT-qPCR) using primers listed in **Table 2**. Gene expression analysis was performed using SYBR Green PCR Master Mix (Roche, Basel, Switzerland), and each sample was analyzed in triplicate. Relative cytokine mRNA expression levels were calculated using the 2^−ΔΔCt^ method, with β-actin as the internal control.

**Table 2:** Primers for Real-time quantitative PCR.

Gene	Primer sequences		Accession number
MyD88	F:5’-TACAGGTGGCCAGAGTG GAA-3'	R:5’-GCAGTAGCAGATAAAG GCATCGAA-3’	NM_010851
NOS2	F:5’-GTTCTCAGCCCAACAAT ACAAGA-3'	R:5’-GTGGACGGGTCGATGT CAC-3’	NM_010927
IL-2	F:5’-CCTCAAGTCCTGCAGGC ATG-3'	R:5’-AGTTCACAGGAATAAC TGAG-3’	NM_008366
IL-4	F:5’-CGGATGCAACGACAATC ACT-3'	R:5’-ACCTTGGAAGCCCTAC AGAC-3’	XM_021214104
IL-6	F:5’-CTGCAAGAGACTTCCAT CCAG-3'	R:5’-AGTGGTATAGACAGGT CTGTTGG-3’	NM_031168
IL-10	F:5’-ACTGCTATGCTGCCTGCT CTTACT-3'	R:5’-ACTGGGAAGTGGGTG CAGTTATTG-3’	NM_010548
IL-12	F:5’-AGCACTCCCCATTCCTAC TTCTC-3'	R:5’-CCCTCCTCTGTCTCCTT CATCTT-3’	NM_001303244
IL-1β	F:5’-TGGAGAGTGTGGATCCC AAGCAAT-3'	R:5’-TGCTTGTGAGGTGCTG ATGTACCA-3’	NM_008361
TGF-β	F:5’-GACTGTCCACTTGCGAC AAC-3'	R:5’-GGCAAACCGTCTCCAG AGTAA-3’	NM_009371
TLR-1	F:5’-TACAGTTCCTGGGGTTG AGC-3'	R:5’-ATTCGGGGTCTTCTTTT TCC-3’	NM_001276445
TLR-2	F:5’-ATCAGTCCCAAAGTCTA AAGTC-3'	R:5’-GGCCAAGTTAGTATCT CTTAGT-3’	NM_021158462
TLR-3	F:5’-GGGGTCCAACTGGAGAA CCT-3'	R:5’-CCGGGGAGAACTCTTT AAGTGG-3’	NM_ 126166
TLR-4	F:5’-TTGTATCGCCTTCTTAGC AG-3’	R:5’-GGTCCAAGTTGCCGTT TC-3’	NM_021297
TLR-5	F:5’-TCAGACGGCAGGATAGC CTTT-3'	R:5’-AATGGTCAAGTTAGCA TACTGGG-3’	NM_017321698
TLR-6	F:5’-GACTCTCCCACAACAGG ATACG-3'	R:5’-TCAGGTTGCCAAATTC CTTACAC-3’	NM_001384171
TLR-7	F:5’-ATGTGGACACGGAAGAG ACAA-3'	R:5’-ACCATCGAAACCCAAA GACTC-3’	NM_ 133211
TLR-8	F:5’-GGCACAACTCCCTTGTG ATT-3'	R:5’-CATTTGGGTGCTGTTG TTTG-3’	NM_ 133212
TLR-9	F:5’-CCTGCCGCTGACTAATCT -3'	R:5’-AAATTGTGGCCTATAC CCTTC-3’	NM_031178
TNF-α	F:5’-CTGAACTTCGGGGTGAT CGG-3'	R:5’-GGCTTGTCACTCGAAT TTTGAGA-3’	NM_013693
Arg-1	F:5’-TGTCCCTAATGACAGCT CCTT-3'	R:5’-GCATCCACCCAAATGA CACAT-3’	NM_007482
NF-κB	F:5’-AGGCTTCTGGGCCTTAT GTG-3'	R:5’-TGCTTCTCTCGCCAGG AATAC-3’	NM_001365067
β-actin	F:5’-GGCTGTATTCCCCTCCAT CG-3'	R:5’-CCAGTTGGTAACAATG CCATGT-3’	NM_007393

### Immunoblot analysis

Total protein was extracted from macrophages in each treatment group using RIPA buffer supplemented with 1% protease inhibitor cocktail (Roche, Basel, Switzerland) according to the manufacturer’s instructions (35). Cells were ultrasonicated and centrifuged at 4°C, 12000 rpm (≈ 16,700 × g) for 10 minutes. The supernatant was mixed with 5× Loading buffer, boiled for 10 minutes, then cooled on ice for 5 minutes, and stored at -20°C. Proteins were separated by SDS-PAGE, transferred to polyvinylidene difluoride (PVDF) membranes (Vazyme, Nanjing, China), blocked with 5% nonfat milk for 2h at room temperature, and incubated with primary antibody overnight at 4°C, followed by HRP-conjugated secondary antibody for 1h at room temperature. Then, protein bands were visualized with the Western Lightning Plus Enhanced Chemiluminescence Kit (Applygen Technologies Inc., Beijing, China) through chemiluminescent detection.

In this study, the primary antibodies used included rabbit anti-mouse TLR2, TLR8, TLR9, NF-κB, and MyD88 monoclonal antibodies (Abcam, Cambridge, UK), as well as mouse anti-His-tag and mouse anti-GFP-tag monoclonal antibodies (Abclonal, Wuhan, China). In addition, a laboratory-generated mouse anti-VP7 monoclonal antibody specific for PoRV JL94 strain was also used in this study. The secondary antibodies were horseradish peroxidase (HRP)-conjugated goat anti-mouse IgG and HRP-conjugated goat anti-rabbit IgG. All antibodies used in this study are listed in **Table 3**, along with their suppliers and catalog numbers.

**Table 3:** Antibody information.

Group	Antibody	Supplier	Catalog information
1	CD45 Monoclonal Antibody (30-F11), FITC, eBioscience™	Thermo Fisher Scientific, Waltham, USA	AB_465050(11-0451-82)
2	CD11b Monoclonal Antibody (M1/70), Alexa Fluor™ 660, eBioscience™	Thermo Fisher Scientific, Waltham, USA	AB_2896245(606-0112-82)
3	F4/80 Monoclonal Antibody (BM8), PE, eBioscience™	Thermo Fisher Scientific, Waltham, USA	AB_465922(12-4801-80)
4	CD64 Monoclonal Antibody (X54-5/7. 1), PE, eBioscience™	Thermo Fisher Scientific, Waltham, USA	AB_2735014(12-0641-82)
5	CD8a Monoclonal Antibody (53-6.7), APC, eBioscience™	Thermo Fisher Scientific, Waltham, USA	AB_469336(17-0081-83)
6	MHC Class I Monoclonal Antibody (IVA26), Alexa Fluor™	Thermo Fisher Scientific, Waltham, USA	AB_2913069(MA5-44137)
7	MHC Class II (I-A/I-E) Monoclonal Antibody (M5/114.15.2), FITC, eBioscience™	Thermo Fisher Scientific, Waltham, USA	AB_465233(11-5321-85)
8	CD80 (B7- 1) Monoclonal Antibody (16- 10A1), FITC, eBioscience™	Thermo Fisher Scientific, Waltham, USA	AB_465135(11-0801-86)
9	CD86 (B7-2) Monoclonal Antibody (GL1), FITC, eBioscience™	Thermo Fisher Scientific, Waltham, USA	AB_465148(11-0862-82)
10	CD3 Monoclonal Antibody (17A2), FITC, eBioscience™	Thermo Fisher Scientific, Waltham, USA	AB_2572431(11-0032-82)
11	CD4 Monoclonal Antibody (CT-CD4), Biotin	Thermo Fisher Scientific, Waltham, USA	AB_2538838(MA5- 17448)
12	CD8b Monoclonal Antibody (eBioH35- 17.2 (H35- 17.2)), FITC, eBioscience™	Thermo Fisher Scientific, Waltham, USA	AB_657764(11-0083-82)
13	TLR6 Polyclonal Antibody	Thermo Fisher Scientific, Waltham, USA	AB_ 10979845(PA5- 11601)
14	Anti-TLR2 antibody	Abcam, Cambridge, UK	AB209217
15	Anti-TLR8 antibody	Abcam, Cambridge, UK	AB180610
16	Anti-TLR9 antibody	Abcam, Cambridge, UK	AB134368
17	Anti-NF-kB p65 (phospho S536) antibody	Abcam, Cambridge, UK	AB76302
18	Anti-MyD88 antibody	Abcam, Cambridge, UK	AB219413
19	Goat Anti-Mouse IgA alpha chain (FITC)	Abcam, Cambridge, UK	AB97234
20	Goat Anti-Mouse IgA alpha chain (HRP)	Abcam, Cambridge, UK	AB97235
21	Goat Anti-Rabbit IgG H&L (HRP)	Abcam, Cambridge, UK	AB6721
22	Goat Anti-Mouse IgG H&L (HRP)	Abcam, Cambridge, UK	AB6789
23	HRP-conjugated Mouse anti His-Tag mAb	Abclonal, Wuhan, China	AE028
24	HRP-conjugated Mouse anti GFP-Tag mAb	Abclonal, Wuhan, China	AE030

### Detection of specific antibody levels

After immunization, blood samples from the retro-orbital venous plexus and fresh fecal samples were collected at 7-day intervals. Serum from the PBS-immunized group served as a negative control. The processing and storage of blood and fecal samples were performed as described above (fecal samples were homogenized and centrifuged to obtain supernatants for analysis).In addition, jejunum tissues were collected from mice at the endpoint (the collection and processing methods were as described above), and the levels of specific IgA antibodies in mouse intestinal homogenates were measured by ELISA.

Serum immunoglobulin G (IgG) and fecal secretory immunoglobulin A (IgA) levels were determined by ELISA using kits purchased from Abcam (Cambridge, UK). The procedure was as follows: VP7 protein was diluted to 2 μg/mL in coating buffer, added to 96-well plates at 100 μL per well, and incubated overnight at 4 °C. After washing, the plates were blocked with 200 μL per well of PBS containing 5% skim milk for 2 h at 37 °C. For IgG detection, serum samples (diluted 1:100 in 5% skim milk) were added at 100 μL per well and incubated for 2 h at 37 °C; for IgA detection, fecal supernatant samples and intestinal supernatant sample (diluted 1:100 in 5% skim milk) were used under the same conditions. After washing, the plates were incubated with HRP-conjugated goat anti-mouse IgG (for serum) or HRP-conjugated goat anti-mouse IgA (for feces and intestine), each diluted to the working concentration in 5% skim milk, for 1 h at 37 °C. After a final wash, 100 μL of TMB substrate solution was added to each well and incubated for 15 min at 37 °C in the dark. The reaction was stopped by adding 50 μL of stop solution, and the absorbance was measured at 450 nm using a microplate reader. All samples were tested in triplicate, and the results were expressed as mean ± SD. The cut-off value was defined as the mean OD450 of the negative control samples plus three standard deviations (mean ± 3 SD). Samples with OD450 values lower than the cut-off value were considered negative. Antibody titers were determined by serial two-fold dilution of the samples, and the highest dilution yielding an OD450 value above the cut-off was defined as the positive titer.

### Mixed lymphocyte reaction

Peritoneal macrophages isolated from naive mice were adjusted to a density of 10^6^ cells/mL. The macrophages were stimulated for 24 h with pCI-PoRV-VP7 (750 ng/mL), *L. casei* BG-based pCI-PoRV-VP7 (5×10^7^ particles/mL, 750 ng/mL), LPS (100 ng/well), BGs (5×10^7^ particles/mL), or an equal volume of PBS (negative control). Before stimulation, peritoneal macrophages were pretreated with 50 µg/mL mitomycin C (Merck, Darmstadt, Germany) at 37 °C for 2 h. After washing with PBS, T lymphocytes (1×10^7^ cells/mL) were co-cultured with the antigen-stimulated macrophages in 96-well plates at lymphocyte-to-macrophage ratios of 1:10, 1:100, and 1:1000. Control groups included lymphocytes alone and macrophages alone, and the blank control wells contained 1640 culture medium. CCK-8 solution (20 µL per well) was added to the 96-well plates, and after incubation at 37 °C for 2 h, the OD450 was measured. The proliferation index was calculated, and the stimulation index (SI) for allogeneic mixed lymphocyte reaction was determined using the formula:SI = (OD sample – OD macrophages only)/(OD T cells only – OD macrophage blank control).

For immunized mice, at the endpoint, splenic lymphocytes were isolated using lymphocyte separation medium and adjusted to a density of 10^7^ cells/mL. The lymphocytes were stimulated with four different antigen conditions: positive control (ConA at 5 µg/mL), blank control (1640 medium), low-antigen group (VP7 at 0.5 µg/mL), and intermediate-antigen group (VP7 at 5 µg/mL). Each condition was set up in quadruplicate wells. The cells were cultured at 37 °C in 5% CO_2_ for 72 h.

T cell proliferation was measured via 3-(4,5-dimethylthiazol-2-yl)-2,5-diphenyltetrazolium bromide (MTT) assay (36).

### Detection of cell surface molecules

Flow cytometry was used to analyze the expression of four surface molecules (MHC-I/MHC-II and CD80/CD86) on macrophages in two settings: (i) on macrophages present in a mixture of peritoneal lymphocytes from naive mice and stimulated macrophages, and (ii) on intestinal macrophages from each group after immunization. The antibodies used are rabbit anti MHC-I-FITC, rabbit anti MHC-II-FITC, rabbit anti CD80-FITC, and rabbit anti CD86-FITC (Thermo fish scientific, Waltham, USA). In addition, used anti-CD3-fluorescein isothiocyanate, anti-CD4-biotin (BIOT, Hangzhou, China), and anti-CD8-phycoerythrin (Sigma, St.Louis, MO, USA) to sift CD4+ and CD8+ T Cells by flow cytometry. All samples were analyzed using BD FACSDIVA software (Becton, Dickinson and Company, New Jersey, USA).

### Detection of intestinal macrophage phagocytic ability

At the endpoint, intestinal macrophages were isolated from mice in each immunization group. The isolated intestinal macrophages were seeded into 24-well plates and cultured in a 37 °C incubator with 5% CO_2_ for 4* h*. Subsequently, 500 µg of dextran-FITC was added to each well. After incubation at 37 °C for 30* min*, the cells were washed three times with PBS. The phagocytic capacity of intestinal macrophages was evaluated by observing the intensity of green fluorescence under a fluorescence microscope.

### Evaluation of intestinal EGFP expression and total IgA production

At the endpoint, intestinal tissues were collected from mice orally immunized with the pCI-EGFP plasmid. The tissues were embedded in Optimal Cutting Temperature (OCT) compound and rapidly frozen. Frozen sections (6 μm thick) were prepared using a pre-cooled cryostat and mounted onto glass slides. After air-drying at room temperature, the sections were fixed with cold acetone at −20 °C. The sections were then air-dried again and stored at 4 °C until use. Fluorescence signals were observed using a laser confocal microscope (Olympus Corporation, Tokyo, Japan).

At the endpoint, intestinal tissues were collected and prepared as frozen sections. The sections were washed three times with PBS for 2* min* each, followed by blocking with 3% BSA for 30* min*. After washing three times with PBS for 1* min* each, the sections were incubated overnight at 4 °C with FITC-conjugated anti-mouse IgA antibody (1:100 dilution). The sections were then washed again three times with PBS for 1* min* each and counterstained with DAPI for 5* min*. Following a final PBS wash (three times, 1* min* each), fluorescence signals were observed using a laser confocal microscope.

Fluorescence intensity was quantified using ImageJ software by measuring the mean fluorescence intensity (MFI) in randomly selected regions of interest (ROI). All imaging parameters were kept constant across groups.

### Immunofluorescence staining

Frozen intestinal tissue sections were prepared following immunization. Sections were washed three times with PBS for 2* min* each. Subsequently, several drops of 3% BSA solution were added, and the sections were blocked for 30* min*. After blocking, sections were washed three times with PBS for 1* min* each wash. FITC-conjugated mouse anti-IgA antibody (1:100 dilution) was applied, and the sections were incubated overnight at 4 °C. The next day, sections were washed three times with PBS for 1* min* each wash. Nuclei were counterstained with DAPI for 5* min*, followed by three additional PBS washes (1* min* each). Finally, the sections were examined using a laser scanning confocal microscope (Olympus Corporation, Tokyo, Japan).

### Intestinal morphology

Seven days post-challenge, mice were euthanized by cervical dislocation under anesthesia, and the abdominal cavity was opened under sterile conditions. The small intestine was carefully excised, and the jejunal segment was identified, gently flushed with cold PBS to remove luminal contents, and longitudinally opened along the mesenteric border. The jejunal tissues were fixed in 4% paraformaldehyde, followed by paraffin embedding and sectioning, and then subjected to hematoxylin and eosin (H&E) staining as previously described (37). Histopathological changes were observed and recorded under a light microscope. Villus length, crypt depth and villus-to-crypt ratio (V/C) were measured using Image-Pro software (Media Cybernetics, Rockville, MD, USA).

### Viral load and neutralization assay

Naked DNA and ghost-loaded DNA were respectively immunized into mice via intramuscular or oral route. At the endpoint, the animals were orally challenged with 200 μL of PoRV (10^5^ TCID50/mL). At 7 days post-challenge, the mice were euthanized by cervical dislocation, and jejunum tissues were collected. The intestinal contents were gently rinsed with pre-chilled sterile PBS. After blotting away surface moisture, the tissues were aliquoted into RNase-free cryotubes, immediately frozen in liquid nitrogen, and subsequently placed in a tissue grinder. Total RNA was extracted using the TRIzol method (as described in the Real-time PCR section), and viral loads were determined by RT-qPCR.

For the virus neutralization test, whole blood was collected from mice via retro-orbital venous plexus blood collection at 7 days post-challenge. The blood was placed into 1.5 mL sterile centrifuge tubes and allowed to stand at 4 °C for 2 h, then centrifuged at 4 °C and 5000 rpm (≈ 5,000 × g) for 20 min. The upper serum layer was carefully aspirated, aliquoted, and stored at −80 °C until use. Prior to the virus neutralization test, the serum samples were heat-inactivated at 56 °C in a water bath for 30 min to remove complement interference. In a 96-well cell culture plate, the sera were subjected to two-fold serial dilutions using serum-free DMEM medium, and then mixed with an equal volume of PoRV virus solution containing 100 TCID50. After incubation at 37 °C for 1 h, the mixture was added to MA-104 cells and incubated for 5–7 days to observe the cytopathic effect (CPE). The serum neutralizing antibody titer (TCID50) was calculated using the Reed-Muench method.

### Statistical analysis

Data are presented as the mean ± standard deviation (SD). The normality of each dataset was assessed using the Shapiro–Wilk test before statistical comparison. For comparisons between two groups, the Mann–Whitney U test was used. For comparisons among more than two groups, one-way analysis of variance (ANOVA) was performed, followed by Tukey’s HSD *post hoc* test for multiple pairwise comparisons or Dunnett’s *post hoc* test when each treatment group was compared with a single control group. Statistical analyses were performed using PSS software (SPSS, Chicago, USA). Significant differences were defined as: *P < 0.05, **P < 0.01, ***P < 0.001; ^a^*P* < 0.05, ^b^*P* < 0.01, ^c^*P* < 0.001.

## Results

### Preparation and characterization of *L. casei* BGs-based DNA vaccines

**Figure 1:**
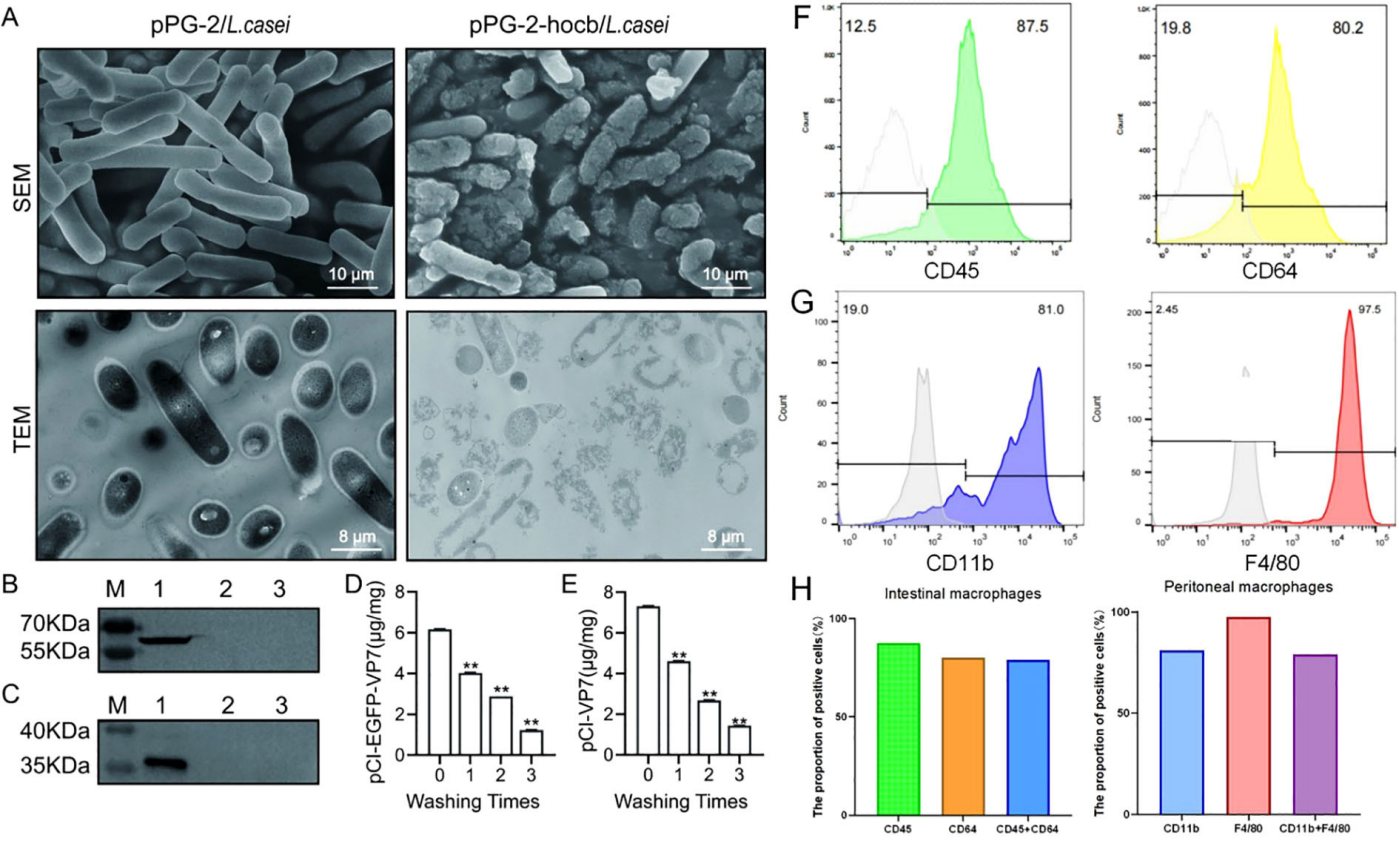
Characteristics of the *L. casei* bacteria ghosts carrying plasmids. **(A)** Transmission electron microscopy (TEM) and scanning electron microscopy (SEM) analyses were conducted on *L. casei 393* following induction. pPG-2/*L. casei* group is control, pPG-2-hocb/*L. casei* group prepares bacterial ghosts by inducing lytic gene expression. **(B, C)** EGFP-VP7 and VP7 protein production were analyzed by western blotting, which resolved EGFP-VP7 as an immunoreactive 62 kD **(B)** band and VP7 as 37kDa **(C)** band. M, marker; 1, 293 cells were transfected with pCI-PoRV-VP7; 2, 293 cells were transfected with pCI-neo; 3, Untreated 293 cells. **(D, E)** RT-qPCR detected EGFP-VP7 **(D)** and VP7 **(E)** DNA content to determine the loading capacity and loading stability of *L. casei* BGs. **(F)** Intestinal macrophages were isolated and analyzed by flow cytometry for the expression of surface markers CD45 and CD64. Left: CD45; Right: CD64. **(G)** Peritoneal macrophages were isolated from mice and analyzed by flow cytometry for the expression of CD11b and F4/80. Left: CD11b; Right:F4/80. **(H)** Proportions of CD45^+^CD64^+^ and CD11b^+^F4/80^+^ dual-positive macrophages in the intestinal and peritoneal compartments. PBS was used as the negative control. *Data are presented as mean ± SD, with significance indicated by **p < 0.01. Statistical significance was determined using one-way ANOVA followed by Dunnett’s *post hoc* test. n = 3 independent animal-derived biological samples.

To verify *L. casei* BGs formation, electron microscopy was employed for morphological analysis. Scanning and transmission electron micrographs showed that induced pPG-2-hocb*/L. casei 393* cells, compared with control cells, exhibited mid-cell or apical perforations while maintaining overall cellular integrity (**Figure 1A**), In this study, PoRV, which targets small intestinal epithelial cells, was selected as the model virus, and a PoRV vaccine was constructed based on its capsid antigen VP7. To confirm that the constructed plasmids were functional, 293T cells were transfected with pCI-EGFP-VP7 or pCI-PoRV-VP7. Immunoblot analysis of cell lysates and culture supernatants confirmed expression of the expected 62 kDa fusion protein and 37 kDa VP7 protein, respectively (**Figures 1B, C**), confirming that the plasmids were functional. The plasmid loading capacity of BGs was quantified by RT-qPCR, showing maximum loading capacities of 7.3 µg/mg for pCI-PoRV-VP7 and 6.17 µg/mg for pCI-PoRV-VP7-EGFP. After washing with PBS, the retained loading capacities were 1.43 µg/mg and 1.21 µg/mg, respectively, indicating that a fraction of the plasmid is retained.These results indicate BGs can encapsulate plasmids (**Figures 1D, E**).

### Isolation and purity of peritoneal and intestinal macrophages

To verify the effect of *L. casei* BGs on macrophages, peritoneal macrophages were isolated from naive mice. Intestinal macrophages were isolated from immunized mice (as described in the immunization protocol). The expression levels of CD45 and CD64 on intestinal macrophages, as well as CD11b and F4/80 on peritoneal macrophages, were detected by flow cytometry. The results are shown in **Figures 1F–H**. In the isolated peritoneal macrophages, the purity of CD11b^+^ F4/80^+^ was > 78%, indicating that peritoneal macrophages of high purity were successfully obtained. For the isolated intestinal macrophages, the positivity rates of the surface markers CD45 and CD64 were approximately 87.5% and 80.2%, respectively, and the proportion of CD45^+^CD64^+^ dual-positive cells was approximately 79%, demonstrating that this method enables the isolation of intestinal macrophages with high purity (**Figures 1G, H**).

### *L. casei* BGs enhances macrophage phagocytosis efficiency and promotes cytokine release

**Figure 2:**
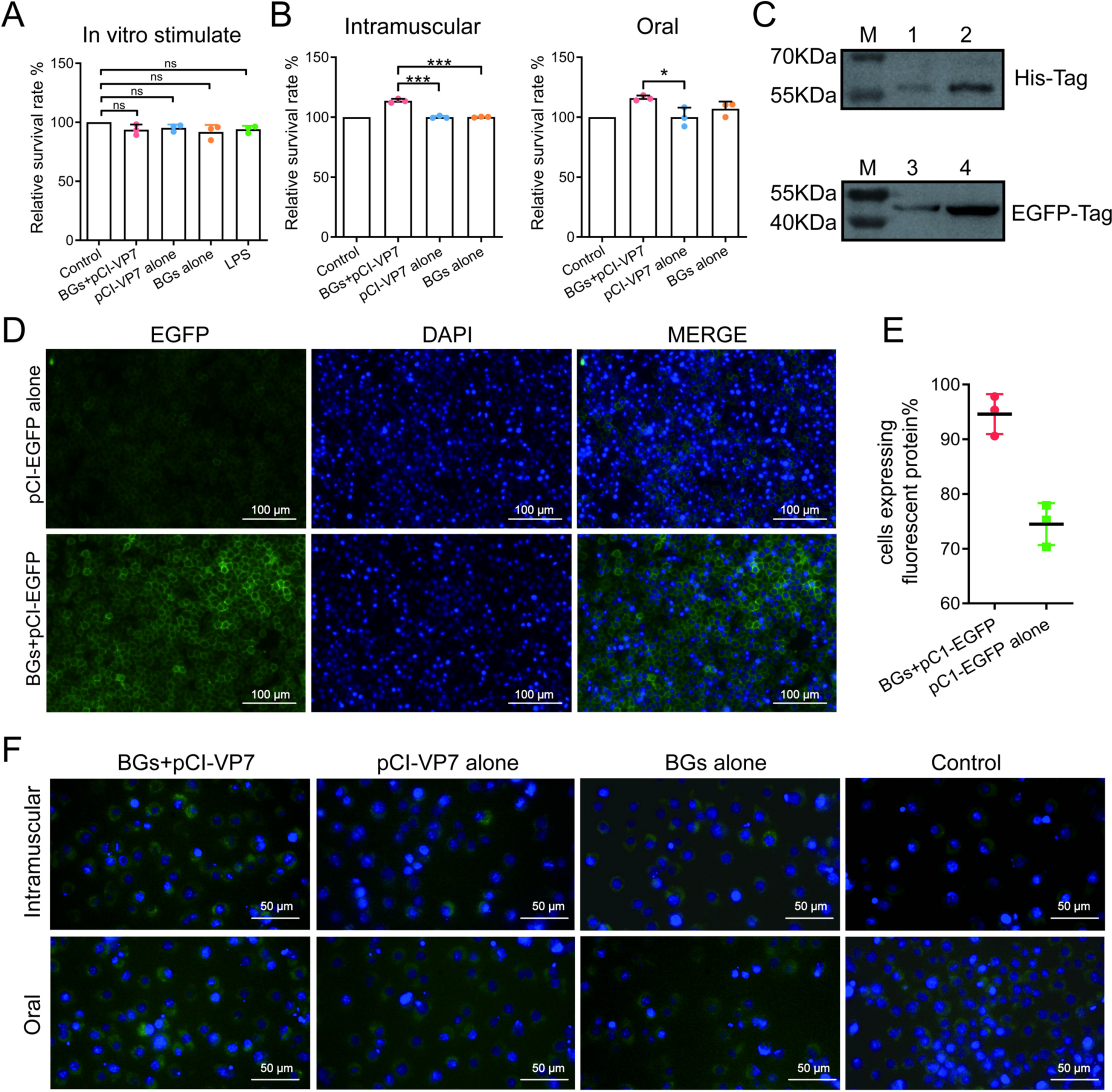
*L. casei* BGs-based DNA vaccines enhanced macrophage phagocytic function. **(A)** Stimulate Peritoneal macrophages with pCI-PoRV-VP7-loaded *L. casei* BGs, pCI-PoRV-VP7, *L. casei* BGs, LPS or PBS, and detect their cell viability using CCK-8 assay. **(B)** Mice intramuscular or orally immunized with pCI-PoRV-VP7-loaded *L. casei* BGs, pCI-PoRV-VP7 or *L. casei* BGs were tested for intestinal macrophage activity using the CCK-8 assay. Control, mice was immunized with PBS. **(C)** Western blot detection of His-VP7 and EGFP-VP7 protein expression levels in intestinal tissue after oral immunization with VP7-loaded *L. casei* BGs or VP7. M, marker; 1, Oral pCI-PoRV-VP7 group; 2, Oral pCI-PoRV-VP7-loaded *L. casei* BGs group; 3, Oral pCI-VP7-EGFP group; 4, Oral pCI-EGFP-PoRV-VP7-loaded *L. casei* BGs group. **(D)** Treat peritoneal macrophages with pCI-EGFP-loaded *L. casei* BGs or pCI-EGFP and observe the green fluorescence intensity during phagocytosis by peritoneal macrophages using a fluorescence microscope. The nuclei were stained with DAPI. **(E)** Detection of fluorescent protein content in mouse peritoneal macrophage phagocytosis loaded with pCI-EGFP plasmid expression by flow cytometry. **(F)** Fluorescence observation of intestinal macrophage phagocytosis of glucan in mice by fluorescence microscope after oral and intramuscular immune. PBS was used as the negative control. *Data are presented as mean ± SD, with significance indicated by *p < 0.05; ***p < 0.001. Statistical significance between two groups was determined using the Mann–Whitney U test. For comparisons among multiple groups, one-way ANOVA followed by Dunnett’s *post hoc* test was used. n = 3 independent animal-derived biological samples.

To verify the effect of *L. casei* BGs-based DNA vaccines on macrophage viability, peritoneal macrophages isolated from naive, non-immunized BALB/c mice were stimulated *in vitro* with pCI-PoRV-VP7-loaded BGs, pCI-PoRV-VP7, BGs, LPS or PBS. CCK8 assay results showed that pCI-PoRV-VP7-loaded BGs did not affect the viability of peritoneal macrophages derived from naive, non-immunized BALB/c mice (**Figure 2A**). Additionally, we observed that after 14 days of oral or intramuscular immunization, the viability of intestinal macrophages in the pCI-PoRV-VP7-loaded BGs group was significantly increased compared with other groups (**Figure 2B**). Subsequently, the phagocytic ability and transfection efficiency of peritoneal and intestinal macrophages were evaluated by fluorescence microscopy, western blotting and flow cytometry. Compared with the pCI-EGFP group, the level of EGFP protein expression in the pCI-EGFP-loaded BGs group was significantly increased (**Figures 2C–E**).

Furthermore, **Figure 2F** shows that the uptake of FITC−dextran by intestinal macrophages in the pCI−PoRV−VP7−loaded BGs group increased approximately twofold compared with that in the pCI−PoRV−VP7 group *in vivo*. These results indicate that the BGs−based DNA vaccine increases FITC−dextran internalization by intestinal macrophages, suggesting enhanced endocytic/macromolecular uptake capacity.

Macrophages exert immunomodulatory functions by releasing cytokines. Therefore, the expression of IL-1β, IL-2, IL-6, IL-12, and TGF-β in the supernatant of peritoneal macrophages stimulated with pCI-PoRV-VP7-loaded BGs, pCI-PoRV-VP7, BGs, LPS and PBS was investigated via ELISA.

**Figure 3:**
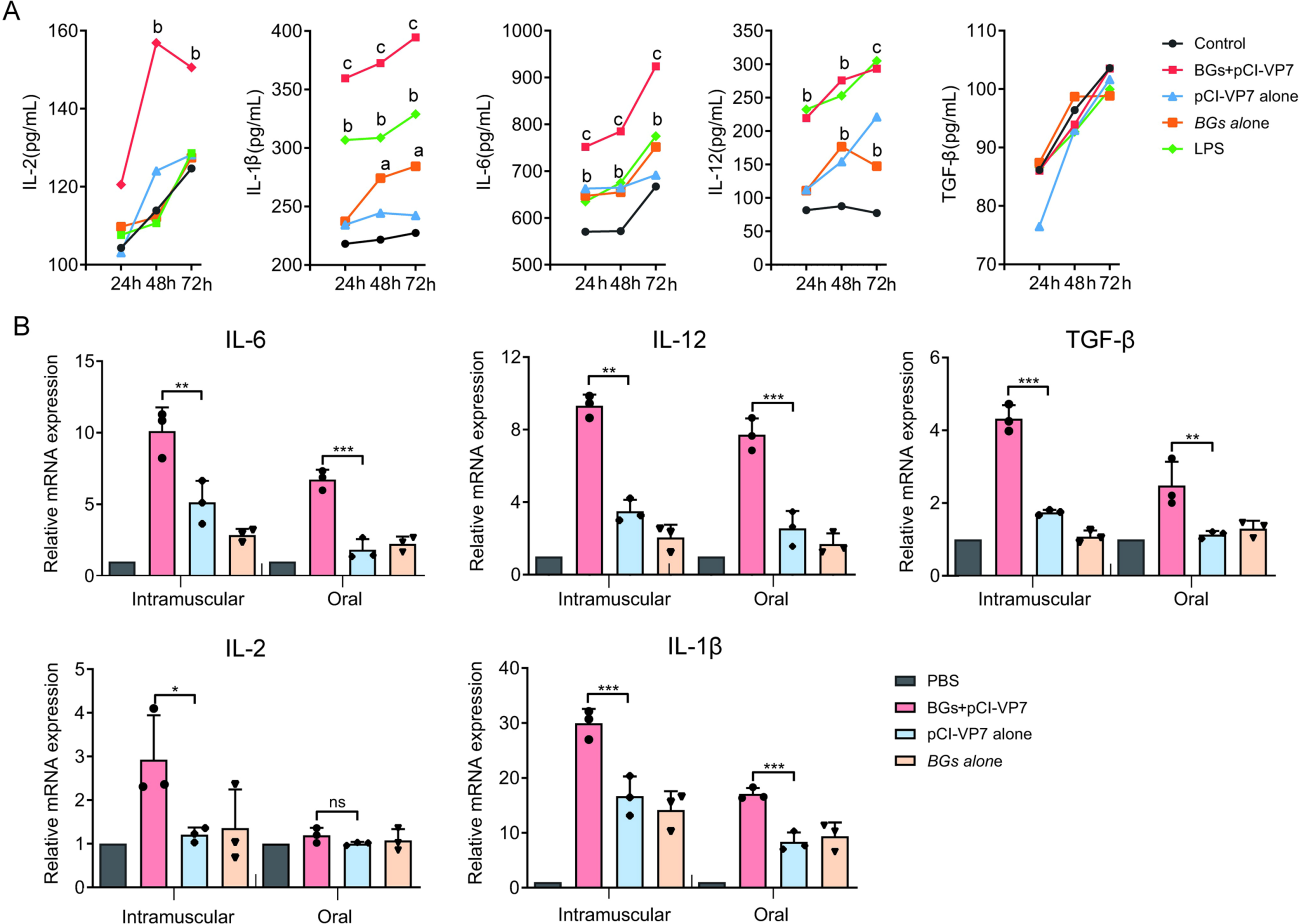
The expression of cytokine after *L. casei* BGs-based DNA vaccine treatment. **(A)** Mice peritoneal macrophages were stimulated with pCI-PoRV-VP7-loaded *L. casei* BGs, pCI-PoRV-VP7, *L. casei* BGs, LPS or PBS for 24h, 48h and 72h. IL-2, IL-1β, IL-6, IL-12 and TGF-β expression levels in the supernatant, all of them were determined by ELISA. **(B)** After intramuscular or oral immunization with pCI-PoRV-VP7-loaded *L. casei* BGs, pCI-PoRV-VP7 or *L. casei* BGs, RT-qPCR detection the intestinal macrophage cytokines mRNA expression levels of IL-2, IL-1 β, IL-6, IL-12, and TGF-β. Significance of differences between the PBS, pCI-PoRV-VP7-loaded *L. casei* BGs, pCI-PoRV-VP7, *L. casei* BGs, groups is represented as follows: a, *p* < 0.05; b, *p*< 0.01; c, *p* < 0.001; *p < 0.05; **p< 0.01; ***p < 0.001. Statistical significance between two groups was determined using the Mann–Whitney U test. For comparisons among multiple groups, one-way ANOVA followed by Dunnett’s *post hoc* test was used. n = 3 independent animal-derived biological samples. PBS was used as the negative control.

As shown in **Figure 3A**, after 24* h* of stimulation, peritoneal macrophages treated with pCI-PoRV-VP7-loaded BGs produced higher levels of IL-1β compared with those treated with pCI-PoRV-VP7. After 48 and 72* h*, the levels of IL-1β, IL-6, and IL-12 were significantly higher in the pCI-PoRV-VP7-loaded BGs group than in the pCI-PoRV-VP7 group (P < 0.01).

Mice were immunized with pCI-PoRV-VP7-loaded BGs, and after 14 days, the cytokine mRNA levels of intestinal macrophages were detected. We also found that the mRNA expression levels of IL-1β, IL-6, IL-12, and TGF-β in intestinal macrophages of the pCI-PoRV-VP7-loaded BGs group were significantly upregulated *in vivo*. Thus the *L. casei* BGs-based DNA vaccine induces upregulation of the secretion of cytokines in macrophages, thereby enhancing their immunomodulatory function.

### *L. casei* BGs promotes M1 polarization of macrophages through the TLR/MyD88/NF-κB signaling pathway

**Figure 4:**
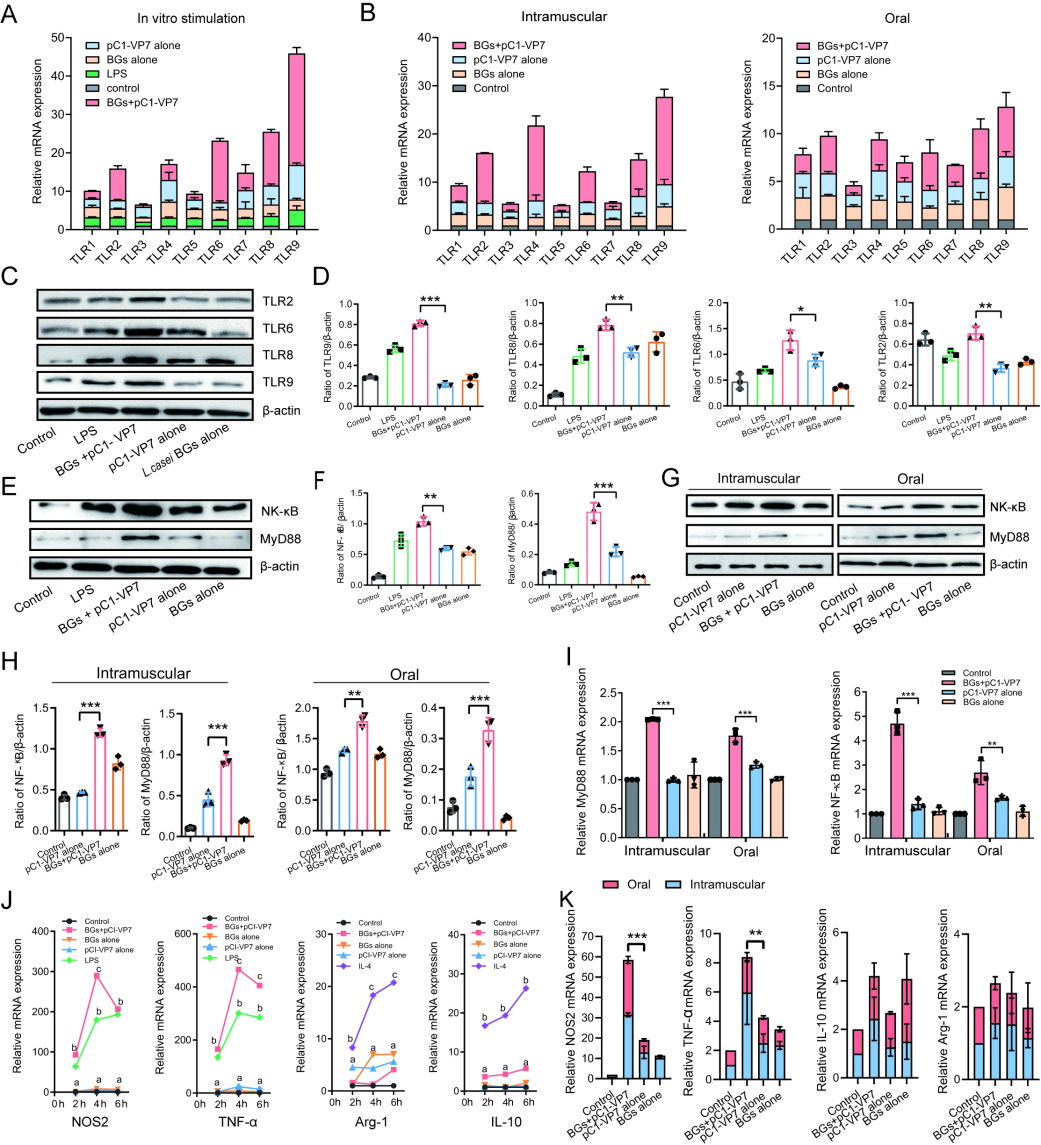
*L. casei* BGs-based DNA vaccines regulates macrophage polarization through TLR/ MyD88/NF-κB Pathway. **(A, B)** The relative mRNA expression of TLR1, TLR2, TLR3, TLR4, TLR5, TLR6, TLR7, TLR8, TLR9 in peritoneal macrophages stimulated *in vitro* **(A)**, as well as in the jejunum of mice immunized by oral or intramuscular injection **(B)**, detected using RT-qPCR. **(C, D)** The contents of TLR2, TLR6, TLR8, and TLR9 in peritoneal macrophages stimulated *in vitro* were determined by Western blot analysis. **(E–H)** The protein expression of MyD88 and NF-κB in peritoneal macrophages stimulated *in vitro* **(E)**, as well as in the jejunum of mice immunized by oral **(G)** or intramuscular injection **(G)**, detected using western blot. **(I)** The relative mRNA expression of MyD88 and NF-κB in the jejunum of mice immunized by oral or intramuscular injection, detected using RT-qPCR. **(J, K)** The relative mRNA expression of NOS2, TNF-α, IL-10, and Arg-1 in peritoneal macrophages stimulated *in vitro* **(J)**, as well as in the jejunum of mice immunized by oral or intramuscular injection **(K)**, was detected using RT-qPCR, with M1-polarization control cells induced by LPS (50 ng/mL) and M2-polarization control cells induced by IL-4 (40 ng/mL). PBS was used as the negative control. Data are presented as mean ± SD from three independent biological replicates, with significance indicated by *p < 0.05; **p < 0.01; ***p < 0.001. Statistical significance between two groups was determined using the Mann–Whitney U test. For comparisons among multiple groups, one-way ANOVA followed by Dunnett’s *post hoc* test was used. n = 3 independent animal-derived biological samples.

M1 macrophages play a crucial role in vaccine-induced immune responses. To investigate whether the *L. casei* BGs-based DNA vaccine induces M1 polarization, we assessed the mRNA and protein expression of TLR family members using RT-qPCR and western blot. *In vitro* experiments revealed that the mRNA and protein expression levels of TLR2, TLR6, TLR8, and TLR9 were significantly higher in the pCI-PoRV-VP7-loaded BGs group compared to the pCI-PoRV-VP7 group, with both groups showing higher expression than other control groups (**Figures 4A, C, D**). Additionally, we analyzed the protein expression of p65 and MyD88, key components of the NF-κB/MyD88 signaling pathway, by western blot. The expression levels of p65 and MyD88 were significantly upregulated in the pCI-PoRV-VP7-loaded BGs group compared to the pCI-PoRV-VP7 group (**Figures 4E, F**).

*In vivo* experiments also demonstrated changes in the mRNA and protein expression levels of TLRs and MyD88/NF-κB (p65). As shown in **Figure 4B**, the mRNA expression levels of TLR1–TLR9 in intestinal macrophages were significantly increased in the pCI-PoRV-VP7-loaded BGs group following oral or intramuscular *injection*, with TLR2, TLR4, TLR6, TLR8, and TLR9 showing the most pronounced upregulation. Furthermore, both oral and intramuscular immunization led to increased MyD88 and p65 mRNA and protein expression in the pCI-PoRV-VP7-loaded BGs group (**Figures 4G–I**).

To further investigate the role of the BGs-based DNA vaccine in macrophage polarization, RT-qPCR was used to assess the mRNA expression levels of intestinal macrophage polarization markers. The results showed that TNF-α and NOS2 mRNA expression levels were significantly increased in the pCI-PoRV-VP7-loaded BGs group compared with the M1-inducing control group. In contrast, IL-10 and Arg-1 expression levels were not significantly altered in the pCI-PoRV-VP7-loaded BGs group and were significantly lower than those observed in the M2-inducing control group (**Figure 4J**). Additionally, intestinal macrophages were isolated from immunized mice, and polarization marker expression was analyzed by RT-qPCR. After both oral and intramuscular *injection*, TNF-α and NOS2 expression levels were significantly upregulated in the pCI-PoRV-VP7-loaded BGs group (**Figure 4K**).

Collectively, these results indicate that the *L. casei* BGs-based DNA vaccine promotes M1 polarization of intestinal macrophages.

### *L. casei* BGs enhances macrophage antigen presentation ability

**Figure 5:**
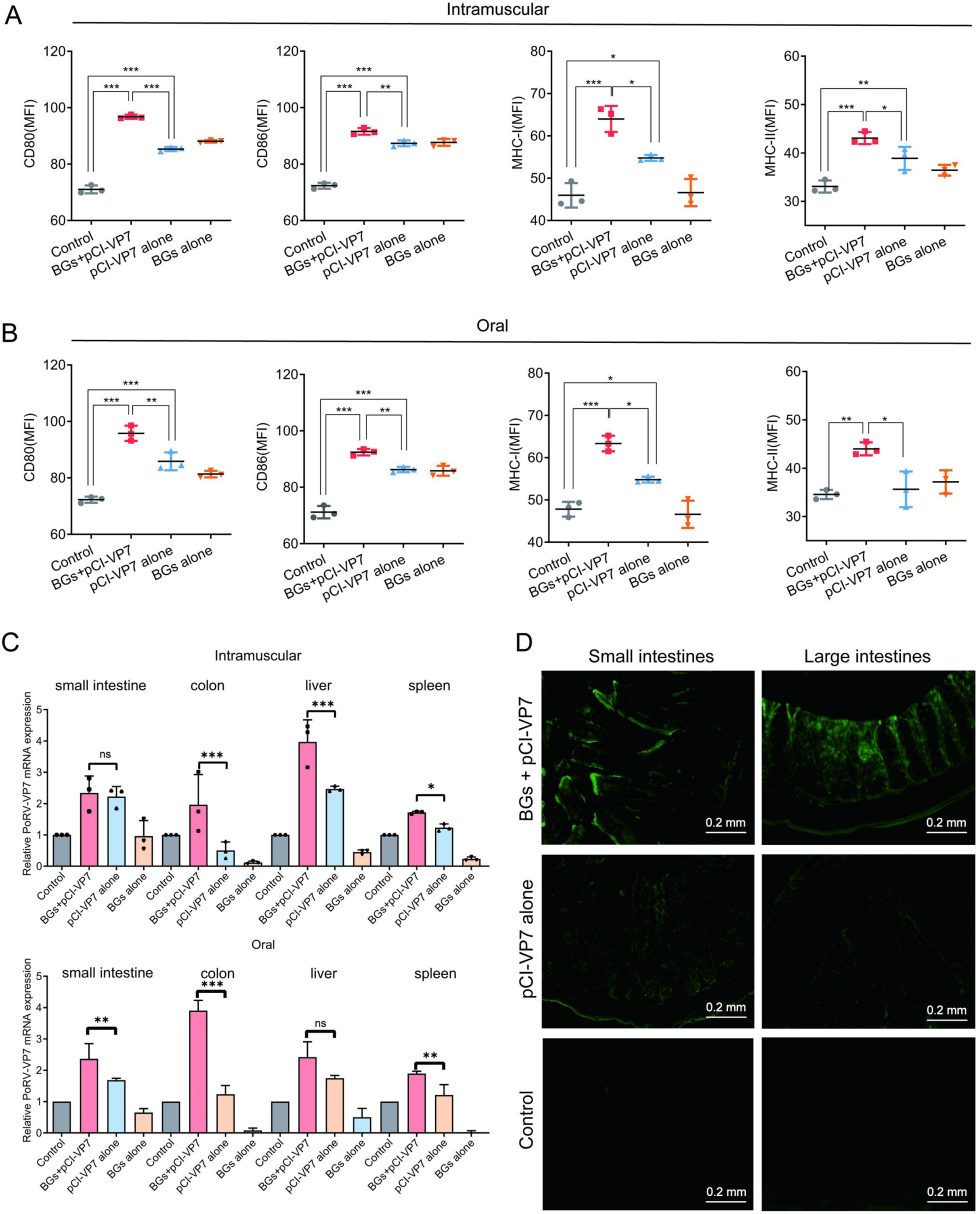
*L. casei* BGs-based DNA vaccines enhances antigen presentation ability of intestinal macrophages. **(A, B)** The expression of CD80, CD86, MHC-I and MHC-II in the Intestinal macrophages of mice immunized through oral **(A)** or intramuscular **(B)** routes were also determined using flow cytometry. **(C)** VP7 mRNA expression in the liver, spleen, small intestine and colon tissues of mice immunized through oral or intramuscular routes were analyzed using RT-qPCR. **(D)** Intestinal tissues were harvested from mice 7 days after oral immunization with naked pCI-EGFP plasmid or bacteria ghost–loaded pCI-EGFP and processed into frozen sections. EGFP expression was visualized by fluorescence microscopy. Scale bar: 100μm. PBS was used as the negative control. Data are presented as mean ± SD, with significance indicated by *p < 0.05; **p < 0.01; ***p < 0.001.Statistical significance between two groups was determined using the Mann–Whitney U test. For comparisons among multiple groups, one-way ANOVA followed by Dunnett’s *post hoc* test was used. n = 3 independent animal-derived biological samples.

To evaluate the impact of DNA-loaded *L. casei* BGs vaccination on intestinal macrophages, we isolated these cells from mice in different treatment groups and analyzed the expression of MHC-I, MHC-II, CD80 and CD86 using flow cytometry. **Figures 5A, B** revealed that both pCI-PoRV-VP7 and pCI-PoRV-VP7-loaded BGs induced significant changes in the expression of these surface markers. Compared to control groups treated with BGs or PBS, mice immunized with pCI-PoRV-VP7-loaded BGs or pCI-PoRV-VP7 alone, whether administered via intramuscular or oral administration, showed markedly higher positive rates of MHC-I, MHC-II, CD80, and CD86. Importantly, the pCI-PoRV-VP7-loaded BGs group showed significantly higher expression levels of these markers compared with the pCI-PoRV-VP7 group. These results suggest that the *L. casei* BGs-based DNA vaccine delivery system enhances the activation and antigen-presenting potential of intestinal macrophages.

To determine whether the DNA-loaded *L. casei* BGs vaccines enhance antigen loading in various tissues of immunized animals, we detected the mRNA expression levels of VP7 in the liver, spleen, small intestine, and colon of mice. After intramuscular *injection*, the mRNA expression levels of VP7 in the pCI-PoRV-VP7-loaded BGs group were significantly increased in the liver, spleen, and colon compared to the pCI-PoRV-VP7 group. Following oral administration, the mRNA expression levels of VP7 in the spleen, small intestine, and colon were also significantly increased in the pCI-PoRV-VP7-loaded BGs group (P<0.05) (**Figure 5C**). These results suggest that BGs-mediated delivery enhances the tissue distribution and expression efficiency of DNA vaccines *in vivo*.

Additionally, after immunizing mice with pCI-EGFP and pCI-EGFP-loaded BGs for 7 days, frozen intestinal sections were prepared, and the expression of fluorescent proteins was observed under a fluorescence microscope. Compared to the pCI-EGFP group, the fluorescence intensity in the intestinal tract was significantly enhanced in the pCI-EGFP-loaded BGs group (P < 0.001) (**Figure 5D**), indicating that *L. casei* BGs can effectively improve *in vivo* transgene expression efficiency of DNA vaccines.

### *L. casei* BGs enhance DNA vaccine efficacy by promoting T cell proliferation and polarization

**Figure 6:**
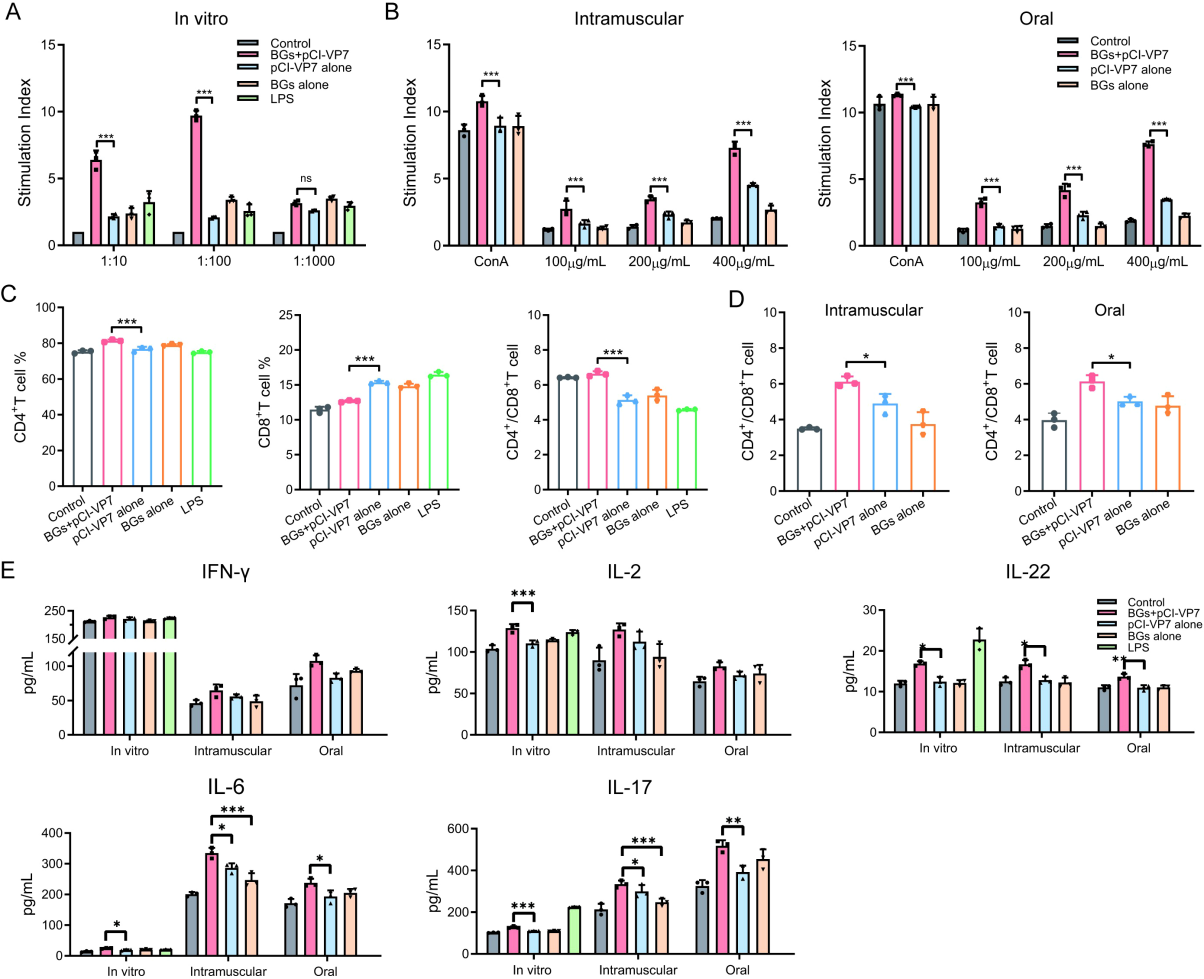
Effect of *L. casei* BGs-based DNA vaccines treatment on lymphocyte proliferation. **(A, B)** CCK-8 method was used to detect lymphocyte proliferation index Lymphoproliferation assay. The effects of different *in vitro* treatment groups on the proliferation of lymphocytes after co-culture with peritoneal macrophages and spleen cells **(A)**. Lymphocytes from the mice immunized by the intramuscular **(B)**, or oral **(B)** route were prepared at the endpoint and cultured with VP7 protein of final concentration of 0.5 or 5 mg/mL. **(C)** After mixing macrophages stimulated *in vitro* with splenic lymphocytes, the CD4^+^ and CD8^+^ T lymphocyte populations of mice were analyzed using flow cytometry, and the CD4^+^/CD8^+^ ratio was calculated. **(D)** After intramuscular and oral immunization of mice, the CD4^+^/CD8^+^ ratio in splenic lymphocytes was detected by flow cytometry. **(E)** ELISA method was used to detect the secretion levels of cytokines IFN-γ, IL-2, IL-17, IL-6 and IL-22 in lymphocyte mixing experiments. PBS was used as the negative control. Data are presented as mean ± SD from three independent biological replicates, with significance indicated by *p < 0.05; **p < 0.01; ***p < 0.001.Statistical significance between two groups was determined using the Mann–Whitney U test. For comparisons among multiple groups, one-way ANOVA followed by Dunnett’s *post hoc* test was used. n = 3 independent animal-derived biological samples.

To investigate whether macrophages treated with DNA-loaded *L. casei* BGs promote lymphocyte proliferation, a CCK-8 assay was used to determine the stimulation index. The results showed that the pCI-PoRV-VP7-loaded BGs group significantly enhanced T cell proliferation compared with other groups. When the ratio of peritoneal macrophages to splenocytes was 1:100, the lymphocyte proliferation index in the pCI-PoRV-VP7-loaded BGs group reached the highest level among all groups (**Figure 6A**). *In vivo* experiments further demonstrated that splenic lymphocyte proliferation was significantly increased in the pCI-PoRV-VP7-loaded BGs group, with the highest proliferation index observed at 400 µg/mL of VP7 protein following both oral and intramuscular *injection* (**Figure 6B**). These findings suggest that macrophage activation induced by BGs-based DNA vaccines enhances downstream T cell responses.

After co-culturing splenic lymphocytes with peritoneal macrophages stimulated by different treatment groups, the proportion of CD4^+^ and CD8^+^ T cell subsets were analyzed by flow cytometry. As shown in **Figure 6C**, the pCI-PoRV-VP7-loaded BGs group exhibited a significantly higher percentage of CD4^+^ T cells compared with control groups. The CD4^+^/CD8^+^ T cell ratio in the pCI-PoRV-VP7-loaded BGs group was significantly greater than that in the pCI-PoRV-VP7 group alone (P<0.001), indicating a stronger induction of CD4^+^ T cell-biased response. It should be noted that while a higher CD4^+^/CD8^+^ ratio may be beneficial for enhancing helper T cell activity in certain immune contexts, maintaining a balanced CD4^+^/CD8^+^ ratio may also be desirable for immune homeostasis and proper regulation.

Additionally, splenic lymphocytes were isolated from mice after either intramuscular or oral administration, and the percentages of CD4^+^ and CD8^+^ T cells were analyzed. As shown in **Figure 6D**, the CD4^+^/CD8^+^ T cell ratio in the pCI-PoRV-VP7-loaded BGs group was significantly higher than that in the pCI-PoRV-VP7 group (P<0.05). These findings suggest that BGs, when used as a DNA vaccine delivery system, enhance T cell-mediated immune responses *in vivo*, thereby contributing to improved immunogenicity.

To further investigate the regulation of T lymphocyte differentiation by macrophages under stimulation with the pCI-PoRV-VP7-loaded BGs vaccine, we measured the concentrations of IFN-γ, IL-2, IL-6, IL-17 and IL-22 in lymphocyte culture supernatants using ELISA kits. Compared with the pCI-PoRV-VP7 group, the levels of IL-2, IL-6, IL-17 and IL-22 were significantly increased in the pCI-PoRV-VP7-loaded BGs group (P < 0.05). After both intramuscular and oral administration, the serum levels of IL-6, IL-17 and IL-22 in the pCI-PoRV-VP7-loaded BGs group were significantly higher than those in the pCI-PoRV-VP7 group (P < 0.05) (**Figure 6E**). All experimental groups showed higher levels than the PBS group, indicating that the bacterial ghost-loaded DNA vaccine can more effectively promote Th17-related and Th22-related immune responses in the host.

### *L. casei* BGs-based VP7 vaccine enhances humoral and mucosal immunity with robust intestinal protection against PoRV

**Figure 7:**
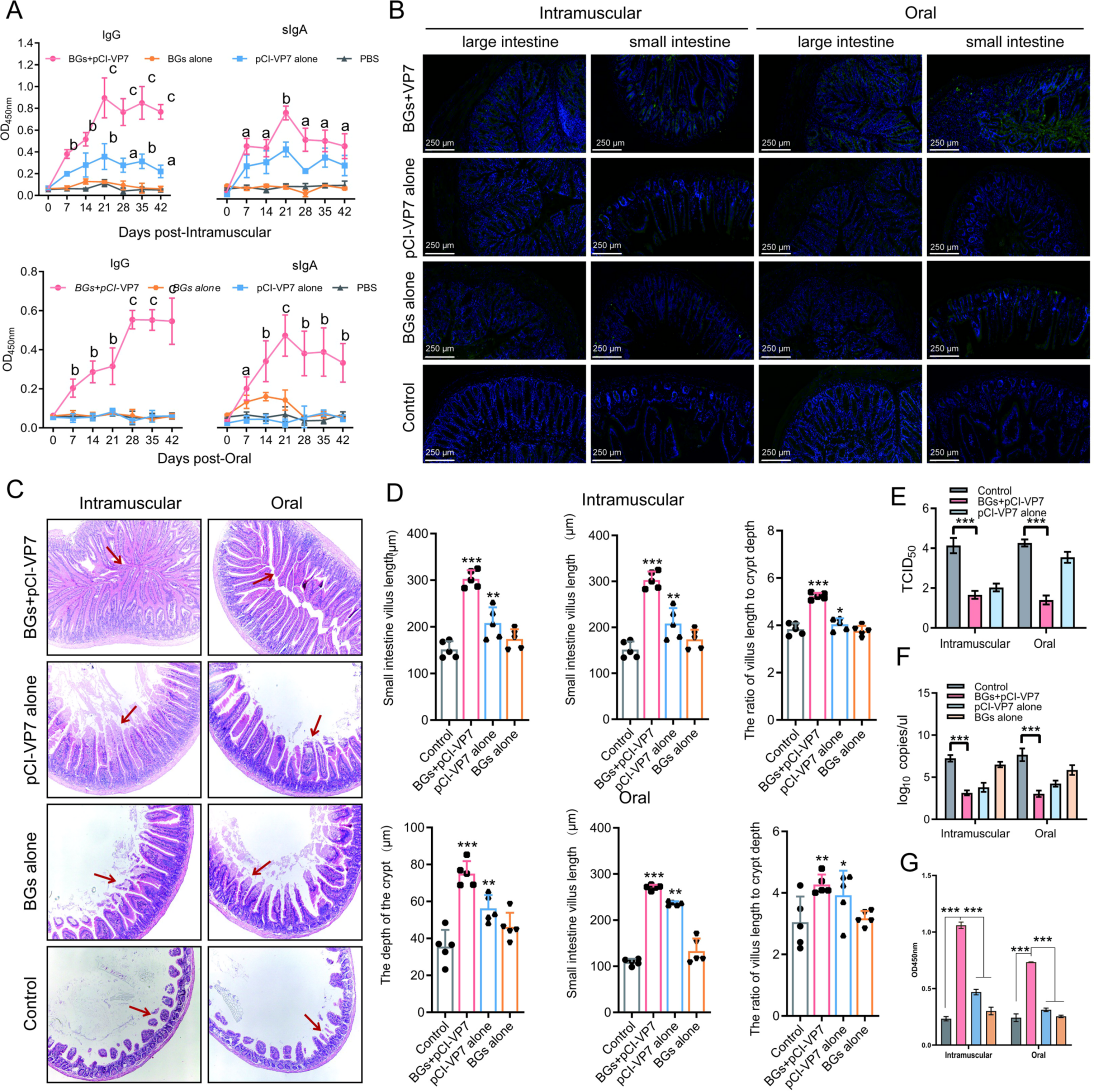
Effect of *L. casei* BGs as a DNA vaccine packaging carrier on immune efficacy. **(A)** The levels of specific antibodies IgG and IgA in the serum were detected by ELISA at 0, 7, 14, 21, 28, 35, and 42 days in intramuscular and oral immunization. **(B)** Expression of IgA in the large and small intestine of mice after intramuscular or oral immune. **(C)** Intestinal morphology of jejunum shown by H&E staining and scanning electron microscope; red arrows indicate sites of intestinal villus damage. **(D)** Analysis of villus length, crypt depth, and the villus length-to-crypt depth ratio. **(E)** The TCID50 titers in serum were measured by virus neutralization test at 7 days post-infection in mice immunized via intramuscular or oral route. **(F)** The viral load (LOG10 copies) in jejunum tissues was measured by qPCR at 7 days post-infection in mice immunized with via intramuscular or oral route. **(G)** Expression of IgA in the jejunum tissues of mice after intramuscular injection or oral immunization. PBS was used as the negative control. Data are presented as mean ± SD from three independent biological replicates, with significance indicated by a, *p* < 0.05; b, *p*< 0.01; c, *p* < 0.001; *p < 0.05; **p< 0.01; ***p < 0.001.Statistical significance between two groups was determined using the Mann–Whitney U test. For comparisons among multiple groups, one-way ANOVA followed by Dunnett’s *post hoc* test was used. n = 3 independent animal-derived biological samples.

To determine the advantages of the *L. casei* BGs-based DNA vaccine in activating immune functions, serum IgG and stool IgA levels were evaluated by ELISA. Following both intramuscular and oral administration, we observed that the production of specific IgG was significantly increased in the pCI-PoRV-VP7-loaded BGs group compared with the pCI-PoRV-VP7 group at most time points. Additionally, IgG levels in both groups were higher than those in the other groups. Notably, oral administration of pCI-PoRV-VP7 alone did not induce specific IgG production in mice (**Figure 7A**). These results suggest that BGs-based DNA vaccine can induce strong humoral immune responses. Next, the levels of specific IgA in the stools of mice immunized via oral or intramuscular routes were measured every seven days. As shown in **Figure 7A**, the level of specific IgA in the pCI-PoRV-VP7-loaded BGs group was significantly higher than in the pCI-PoRV-VP7 groups. Furthermore, oral administration of pCI-PoRV-VP7-loaded BGs induced higher levels of IgA production compared with intramuscular immunization(unpaired t-test, P = 0.023), indicating that oral administration of the DNA-loaded BGs vaccine significantly enhances mucosal immune responses.

To further investigate the impact of *L. casei* BGs-based DNA vaccine on mucosal immunity, total IgA expression in the intestinal mucosa was analyzed using frozen sections. In the oral administration group, the intensity of IgA secretion in the intestinal mucosa was significantly higher in the pCI-PoRV-VP7-loaded BGs group than in the other groups. However, no significant differences were observed among the groups following intramuscular *injection* (**Figure 7B**). These findings suggest that oral administration of DNA-loaded BGs vaccine significantly enhances mucosal immune responses. Additionally, we assessed the protective effect of the BGs-based pCI-PoRV-VP7 vaccine on intestinal tissue against PoRV infection. Following PoRV infection, jejunal tissues were collected for histopathological examination. As shown in **Figures 7C, D**, mice in the pCI-PoRV-VP7-loaded BGs group exhibited longer and more intact villi, deeper crypts, a higher villus length-to-crypt depth ratio with the PBS and pCI-PoRV-VP7 groups. The red arrows in **Figure 7C** highlight areas of villus damage (breakage and loss of integrity), which were prominent in the PBS and pCI-PoRV-VP7 groups but almost absent in the BGs-based vaccine group. In addition, we further determined the viral load in mouse jejunum tissues and performed a virus neutralization test, with the results shown in **Figures 7E, F**. Mice immunized with pCI-PoRV-VP7-loaded BGs exhibited significantly lower jejunal viral loads (**Figure 7E**) compared to the pCI-PoRV-VP7 group, as well as higher serum neutralizing antibody titers (**Figure 7F**). In **Figure 7G**, regardless of the route of immunization (oral or intramuscular), analysis of jejunum tissues collected from mice 14 days after the final immunization revealed that the levels of IgA secretion in the intestinal mucosa of the BGs group carrying pCI-PoRV-VP7 were significantly higher than those in the other groups, consistent with the results shown in **Figure 7A**. These results indicate that the *L. casei* BGs-based DNA vaccine provides immune protection to intestinal tissue after immunization.

## Discussion

As one of the largest immune compartments in the body, the intestinal mucosal immune system serves not only as the first line of defense against pathogen invasion but also as a critical barrier to gastrointestinal pathogens (38–40). DNA vaccines can express antigens within the intestinal mucosa and, after uptake by M cells and APCs, induce both local and systemic immune responses (41, 42). However, naked DNA vaccines are inefficiently taken up, expressed, and presented by APCs, making delivery efficiency within the intestinal mucosa a key factor limiting their efficacy.

In recent years, Lactic acid bacteria have emerged as promising delivery vehicles for DNA vaccines, and the *L. casei* BGs strain exhibits distinct advantages. Here, we successfully constructed *L. casei* BGs capable of stably loading DNA plasmids, as confirmed by scanning and transmission electron microscopy, and immunoblot analysis verified expression of EGFP-VP7 and VP7 proteins in both cell lysates and supernatants. However, this study lacks direct evidence for the *in vivo* translocation of orally administered *L. casei* BGs across the intestinal epithelial barrier and their delivery of DNA to lamina propria immune cells. Although *in vitro* and *in vivo* functional data indirectly support the delivery capacity of this vaccine, the precise trafficking pathway of BGs in the intestine and their interaction with M cells, Peyer’s patches, mesenteric lymph nodes, lamina propria macrophages, and dendritic cells remain unclear. Future studies using fluorescently labeled BGs and labeled plasmids should perform *in vivo* co-localization analyses to elucidate the exact mechanism of action.

To further evaluate vaccine performance, we assessed its immunological effects following intramuscular or oral immunization. The DNA-loaded BGs vaccine significantly increased the expression of MHC-I, MHC-II, CD80, and CD86 in intestinal macrophages. Concurrently, VP7 mRNA levels in tissues and EGFP fluorescence intensity in the intestine were markedly elevated, indicating enhanced antigen expression *in vivo*. Together, these findings demonstrate that *L. casei* BGs improve antigen delivery and presentation efficiency, thereby enhancing the overall immunogenicity of DNA vaccines.

Macrophages play a key role in initiating intestinal mucosal immune responses (43). They reside in the subepithelial layer of the intestinal mucosa, forming an immune barrier that continuously monitors and eliminates pathogens (44). By recognizing and engulfing pathogens that cross the intestinal mucosal barrier, macrophages process and present antigens to T lymphocytes and indirectly facilitate B cell activation, thereby inducing specific immune responses (45). Macrophages present antigens to T lymphocytes via MHC-I and MHC-II molecules, while the costimulatory molecules CD80 and CD86 provide the signals required for T cell activation (46, 47). Roberto et al. reported that following *Trypanosoma* infection, IFN-γ-activated macrophages polarize toward the M1 phenotype and enhance antigen processing and presentation through MHC-I and MHC-II, thereby promoting lymphocyte responses (48). In the present study, *in vitro* and *in vivo* results demonstrated that the DNA vaccine based on the *L. casei* BG strain upregulated the expression of CD80, CD86, MHC-I, and MHC-II on macrophages. This was associated with increased T cell proliferation, higher CD4^+^ T cell percentage and CD4^+^/CD8^+^ ratio, and induction of Th17-related and Th22-related immune responses, thereby enhancing T cell immunity.

The abundance and functional status of macrophages directly influence the efficacy of DNA vaccines (49). Our study found that the DNA vaccine loaded with the *L. casei* BG strain not only did not adversely affect macrophage viability but also significantly enhanced their phagocytic capacity, promoted the release of multiple cytokines, and increased the expression of macrophage maturation and activation markers compared with other experimental groups. Activated macrophages mediate innate and adaptive immunity by secreting cytokines such as interferons and interleukins (50). Sudi et al. reported that macrophages stimulated with verbascose exhibited significantly increased expression of IL-6, IL-1β, IFN-α, and IFN-γ, highlighting verbascose’s immunomodulatory potential (51).

On this basis, our study demonstrated that BGs-based DNA vaccines significantly upregulated the expression of IFN-α, IFN-β, IL-1β, IL-6, and IL-12 in macrophages *in vitro*. Consistently, *in vivo* experiments revealed significantly increased mRNA expression of these cytokines in intestinal macrophages following immunization.

Collectively, these findings suggest that *L. casei* BGs-loaded DNA vaccines regulate intestinal mucosal immunity by promoting macrophage activation and enhancing cytokine-mediated immune signaling.

In addition, macrophage polarization is an important mechanism in regulating immune function (52). Macrophages differentiate into distinct functional phenotypes in response to the microenvironment, microbial stimulation, and cytokine signaling (53), similar to the specialization process of helper T cells. TLRs play a central role in macrophage polarization and activation (54), and their subtypes regulate macrophage function through coordinated transcriptional networks (55). Wang et al. reported that a combination of 14 probiotic strains activated the TLR/MyD88/NF-κB signaling pathway and regulated macrophage polarization by upregulating TLR2, TLR4, TLR6, and TLR9 (56). In line with these findings, our study demonstrated that the *L. casei* BGs-based DNA vaccine also activated the TLR/MyD88/NF-κB pathway, as evidenced by increased expression of TLR2, TLR6, TLR8, and TLR9. This activation may contribute to the enhanced immunogenicity observed for this vaccine platform. However, residual bacterial genomic DNA in the BG preparations was not quantified, and DNase-treated BGs were not included as a control; therefore, the potential contribution of residual bacterial DNA to TLR9 activation cannot be excluded.

To further characterize macrophage responses, we examined polarization markers and found that M1-associated genes (TNF-α and NOS2) were significantly upregulated in the BGs-based DNA vaccine group, whereas M2 markers remained largely unchanged. These results suggest that this vaccine tends to promote M1-like macrophage polarization. M1 macrophages are classically activated cells that play critical roles in pro-inflammatory responses, antimicrobial defense, and the enhancement of phagocytosis and antigen presentation (57). However, in BALB/c mice, VP7-specific IgG subclass profiling, particularly the IgG2a/IgG1 ratio, is commonly used as an important indicator of Th1/Th2 immune bias; therefore, the absence of IgG1 and IgG2a measurements limits our ability to definitively determine Th1-associated polarization in this study. We demonstrate that this vaccine *tends to* promote intestinal macrophage polarization toward an M1-like phenotype, potentially through activation of the TLR/MyD88/NF-κB signaling pathway, thereby contributing to improved immunological efficacy compared with conventional DNA vaccines. Similarly, Hao K et al. reported that immunization of mice with DNA vaccine-loaded *E. coli* DH5α bacterial ghosts resulted in a 90% survival rate following grass carp reovirus infection, whereas the naked DNA vaccine group showed lower protection (58). However, *E. coli*-derived BGs are based on a Gram-negative bacterial platform and may contain LPS-related components, which could raise additional safety concerns. More recently, Yue et al. developed a live probiotic−based oral vaccine platform with promising safety profiles in preclinical models (28). From a different perspective, our non-living *L. casei* BG carrier, derived from a GRAS Gram-positive probiotic, inherently avoids viability-related concerns and thus provides a safer oral vaccine delivery platform with potential biosafety benefits tailored to specific applications.

In sections from our rotavirus challenge model, the intestinal villi of control mice exhibited irregularities (villous atrophy, blunting, and fusion); the naked DNA vaccine group showed similar or milder irregularities, whereas mice immunized with the DNA vaccine loaded with *L. casei* BGs showed no obvious pathological changes.

VP7 is the major outer capsid protein of PoRV and plays a critical role in viral attachment to and entry into host cells. As the primary target of neutralizing antibodies, VP7 is widely recognized as a core protective antigen for the development of anti-rotavirus vaccines. In this study, we evaluated the protective efficacy of a VP7-expressing DNA vaccine delivered by *L. casei* BGs against PoRV challenge. The significantly reduced viral load in ileal tissues and the elevated serum neutralizing antibody titers compared to the naked DNA vaccine group indicate that *L. casei* BGs as a delivery vector can enhance the immunogenicity of the VP7 antigen, thereby inducing a more potent humoral immune response. Histopathological findings further supported this conclusion—the intestinal mucosal structure in the vaccine group remained largely intact, suggesting good mucosal protective capability of the vaccine. Collectively, these results indicate that the protective effect of the pCI-PoRV-VP7-loaded BGs DNA vaccine against PoRV infection is associated with macrophage activation and enhanced antigen presentation-related immune responses, although direct evidence for macrophage-dependent protection requires further validation. One limitation of this study is that some animal-derived endpoints were analyzed using a relatively small number of biological samples, which may limit the statistical power of these analyses. Future studies with larger cohorts are therefore needed to further confirm the immunogenicity, protective efficacy, and safety of this vaccine strategy. These findings provide valuable insights for the development of promising oral mucosal vaccines.

Several limitations should be acknowledged when interpreting the findings of this study. First, only a single immunization dose was tested (50 μg of plasmid DNA combined with 6.8mg BGs). Consequently, whether the observed protective effects are dose−dependent or whether they would be maintained at lower doses remains unclear. This constitutes a key limitation of the present work. Second, owing to limited sample availability and experimental constraints, several recommended control groups were absent from the present study, including empty pCI−neo vector−loaded BGs, irrelevant antigen plasmid−loaded BGs, free plasmid mixed with BGs, DNase−treated BGs preparations, and a live *L. casei* comparator. This lack of controls prevents a definitive distinction between specific plasmid delivery and non−specific PAMP−mediated adjuvant effects. In particular, the possibility that the observed immune responses are partially attributable to the vector backbone, the loading/transfection process, or residual bacterial components—rather than solely to VP7−specific immunity—cannot be excluded. Hence, the true contribution of VP7−mediated protection requires further validation in future studies with optimized experimental designs. Third, one limitation of this study is the incomplete formulation characterization. Parameters such as residual live bacteria, residual genomic DNA/protein contamination, plasmid release kinetics, and stability in simulated gastric/intestinal fluids were not evaluated. Fourth, intestinal macrophages were identified using only CD45 and CD64, which does not allow precise discrimination of macrophage subsets. Future studies employing a more comprehensive flow cytometry panel are needed to further define the role of specific intestinal macrophage subsets in vaccine-induced immunity.

Additionally, future work should address formulation characterization (e.g., residual live bacteria, DNA/protein contamination, release kinetics, and stability in simulated gastrointestinal fluids) and incorporate dose-response designs with appropriate empty vector controls (both *in vivo* and *in vitro*) to more rigorously evaluate the efficacy, mechanism of action, and plasmid loading stability of this *L. casei* BG-based DNA vaccine platform.

In summary, this study demonstrates that the *L. casei* BGs-based DNA vaccine effectively enhances immune responses and protective efficacy against infection by modulating innate immune signaling pathways and promoting macrophage activation, providing an important experimental basis for the development of promising mucosal vaccine delivery strategies.

## Data Availability

The raw data supporting the conclusions of this article will be made available by the authors, without undue reservation.
